# Living with pain—a systematic review on patients’ subjective experiences

**DOI:** 10.1186/s13643-025-02953-6

**Published:** 2025-10-06

**Authors:** Maria Christidis, Essam Ahmed Al-Moraissi, Tasnim Miah, Laura Mihasi, Artin Razavian, Nikolaos Christidis, Giancarlo De la Torre Canales

**Affiliations:** 1 Department of Nursing Science, Sophiahemmet University, Stockholm, 11486 Sweden; 2 Department of Neurobiology, Care Sciences and Society, Karolinska Institutet, Box 23400, Huddinge, 14183 Sweden; 3 Department of Oral and Maxillofacial Surgery, Faculty of Dentistry, Thamar University, Thamar, Yemen; 4 Division of Oral Rehabilitation, Department of Dental Medicine, Karolinska Institutet, Huddinge, 14104 Sweden; 5 Egas Moniz Center for Interdisciplinary Research (CiiEM), Egas Moniz School of Health & Science, Caparica, Almada, Portugal

**Keywords:** Chronic pain, Acute pain, Cancer pain, Patient experiences, Systematic review

## Abstract

**Background:**

Understanding the subjective experiences of patients living with chronic, acute, and cancer pain can significantly enhance the selection of treatment approaches, care, and support, ultimately improving their quality of life. This qualitative systematic review aimed to analyze if the patients’ subjective experiences of living with pain differ between acute, chronic, and cancer pain states.

**Methods:**

After registration in PROSPERO (CRD42023491745), an electronic search was conducted in the databases Medline (Ovid), Embase (embase.com), Cochrane (Wiley), Web of Science (Clarivate Analytics), and CINAHL (EBSCO) from their inception to 19 April 2024. Out of 8443 articles, 62 articles were included. The inclusion criteria that were applied were as follows: (1) participants aged 18 or older; and (2) participants’ subjective experiences of chronic, acute, or cancer pain. The exclusion criteria were as follows: (a) studies presented in other languages than English, Spanish, Portuguese, Greek, and Scandinavian languages; (b) editorials, letters, legal cases, case series, and case-control studies; (c) studies and articles based on duplicated data; (d) study population with ages below 18 years. Forty-four articles regarding chronic pain, thirteen regarding cancer pain, and five regarding acute pain were included. Methodological limitations were assessed using the CASP tool for quality appraisal in qualitative evidence synthesis. Certainty of evidence was assessed with GRADE-CERQual. All included studies showed moderate (*n* = 18) to high (*n* = 44) confidence.

**Results:**

Based on the qualitative synthesis in GRADE-CERQual, four main themes were identified: (1) impact of pain on social life, work life, and family life; (2) challenges in healthcare access; (3) psychological impact and emotional struggles from pain; and (4) barriers to effective pain management.

**Conclusions:**

Taken together, patients with chronic, acute, or cancer pain face challenges in social, work, and personal lives. They often lack recognition and support from healthcare providers, relying on self-managed methods and facing barriers to effective management. Therefore, future research examining how the different pain types affect the lives of the patients and at the same time exploring personalized and collaborative treatment approaches is warranted. In conclusion, patients’ experiences of living with pain remain unexplored in clinical practice. Understanding the impact of various pain types on mental health, self-esteem, daily life, and relationships is crucial. Also, how personalized treatments, collaborative healthcare access, and long-term management strategies can improve quality of life for patients living with pain.

**Systemic review registration:**

PROSPERO CRD42023491745

**Supplementary Information:**

The online version contains supplementary material available at 10.1186/s13643-025-02953-6.

## Background

The International Association for the Study of Pain (IASP) defines pain as “an unpleasant sensory and emotional experience associated with, or resembling that associated with, actual or potential tissue damage” [1]. Pain signals physical injury but can also occur without clear damage. Pain levels and disability vary among individuals, as do responses to treatments [2]. Pain is shaped by biological, psychological, and social factors. Genetics and gender influence sensitivity, while personal experiences, beliefs, and past traumas also play roles [3].

Acute pain is a sudden, short-term experience often with a known cause. Typically lasting up to seven days, it can extend to 30 days [4]. Despite advances in pain management, 80% of post-surgical patients report acute pain, with 86% experiencing moderate to severe levels [5]. Untreated acute pain can cause complications such as deep vein thrombosis, muscle spasms, stress responses, anxiety, and chronic pain [6].

A bidirectional relationship exists between sleep, anxiety, and acute pain. Anxiety and sleep deprivation lower the pain threshold, creating a cycle of pain catastrophizing and avoidance behaviors that increase disability [7]. Early intervention is crucial, as even short-term pain can create long-lasting pain memories, leading to chronic pain [8].

Chronic pain persists beyond 3 months, causing emotional distress and functional impairment [1]. It results from diseases such as fibromyalgia and arthritis or is caused by trauma and injury. Chronic pain affects 19% of European adults, with prevalence estimates ranging from 11 to 40% [3, 9, 10]. Despite frequent doctor visits, many patients do not receive adequate care [10]. Within the chronic pain category, specific conditions such as neuropathic pain, fibromyalgia, and arthritis present distinct clinical and psychosocial profiles. For instance, fibromyalgia is characterized by widespread musculoskeletal pain, central sensitization, fatigue, and elevated rates of anxiety and depression [11]. Neuropathic pain, on the other hand, is associated with nerve damage, burning or shooting sensations, and poorer treatment responses [12]. Arthritis-related pain typically involves localized joint inflammation and mobility limitations, with different quality-of-life impacts compared to fibromyalgia or neuropathic pain [13].

Chronic pain significantly impacts mental health and daily life, limiting activities and social interactions [14]. Depression, anxiety, stress, and poor concentration are common [9]. Higher anxiety levels correlate with lower pain tolerance [15]. Sleep quality also worsens, increasing pain sensitivity [16, 17]. These effects contribute to social isolation and, in some cases, increased suicide risk.

Cancer pain, linked to diagnosis and treatment, affects quality of life. Many patients fear dying in pain more than death itself [18]. Despite treatment advancements, cancer pain remains a major challenge [19]. It affects 51% of patients, with 40% experiencing moderate to severe pain [20]. Cancer-related pain can be divided into: pain directly caused by tumor infiltration; treatment-related pain from surgery, chemotherapy, or radiotherapy; and persistent pain experienced by cancer survivors, such as chemotherapy-induced neuropathy [21].

Post-treatment, cancer survivors often experience chronic pain, depression, and anxiety. Many patients struggle with healthcare access and inadequate pain management information [22, 23]. Enhancing understanding of pain experiences across acute, chronic, and cancer pain states can improve treatment and quality of life. This study aims to systematically review and meta-analyze whether patients’ subjective pain experiences of living with pain differ between acute, chronic, and cancer pain states. We hypothesize that successful pain management requires greater emphasis on patients’ pain experiences and individualized strategies.

## Methods

This systematic review and qualitative meta-analysis was based on observational, cohort, and cross-sectional studies. The review was registered beforehand in the PROSPERO database (the International Prospective Register of Systematic Reviews; with the identification number CRD42023491745) and was performed by the Preferred Reporting Items for Systematic Reviews and Meta-Analyses (PRISMA 2020 checklist) [24], as shown in Table S1.

The research question was formulated according to the PECO method [25], which stands for patients, exposure, comparison, and outcome, making the question easy to understand but also focused on a concise subject. With that in mind, the research's question is the following: In adult individuals (P), subjective experience (O) of living with chronic pain (E) or cancer pain (E) in comparison with acute pain (C).

Population (P): Adult individuals suffering from chronic pain or cancer pain.

Exposure (E): Living with chronic pain or cancer pain.

Comparator (C): Patients suffering from acute pain.

Outcome (O): The subjective experience of patients suffering from chronic- or cancer pain compared to the subjective experience of patients suffering from acute pain.

The inclusion criteria that were applied were as follows: (1) participants aged 18 or older; and (2) participants’ subjective experiences of chronic, acute, or cancer pain. The exclusion criteria were as follows: (1) studies presented in other languages than English, Spanish, Portuguese, Greek, and Scandinavian languages; (2) editorials, letters, legal cases, case series, and case–control studies; (3) studies and articles based on duplicated data; (4) study populations with ages below 18 years.

### Search strategy and study selection

A literature search was performed in the following databases: Medline (Ovid), Embase (embase.com), Cochrane (Wiley), Web of Science (Clarivate Analytics), and CINAHL (EBSCO). The last search was conducted on 2024–04-19.. The search strategy was formulated in Medline (Ovid) in collaboration with the librarian Sabina Gillsund (SG) at the Karolinska Institutet University Library. Relevant Medical Subject Headings (MeSH terms) and free-text keywords were identified for each search topic. The search was subsequently tailored to the other databases, utilizing the Polyglot Search Translator [26] in part. No language restrictions were applied, and databases were searched from their respective inception. Since 1989, when the oldest included study was published, significant developments in pain management services, accessibility, and public awareness have taken place, which may have influenced the patient experiences described in older studies compared to more recent ones. Nevertheless, to provide a comprehensive overview of the existing literature, all studies meeting the inclusion criteria were included. An additional librarian reviewed the strategies before they were executed. Duplicates were removed using the method outlined by Bramer et al. [27], which is an efficient method for duplicate removal involving systematic comparison of reference lists and Digital Object Identifiers (DOIs), enhancing transparency and replicability in systematic reviews. A snow-ball search was conducted to check references and citations of eligible studies from the included studies, but no additional studies were identified [28]. The complete search strategies for all databases are available in Table S2.

The systematic review management platform Rayyan was used to avoid any risk of bias in the process of screening [29]. This was done by two of the authors (TM and LM) independently and blinded from each other, who screened each title and abstract. In cases of disagreement regarding potentially eligible studies, the author AR resolved the conflict, thus acting as a judge. When all disagreements were resolved, the authors (TM and LM) attempted to retrieve the full texts of the included and potentially eligible studies. All retrieved studies were then reviewed in full text by the same authors (TM and LM), independently and blinded from each other, to determine whether they aligned with the inclusion criteria or not. This was done using the Microsoft Office 365 software Excel. Any conflict was resolved by discussion with the author AR.

### Certainty of evidence and critical appraisal

Methodological limitations were assessed independently by two of the authors (TM and LM), after guidance by the author MC, using the critical appraisal skills program (CASP) for quality appraisal in qualitative evidence synthesis. The CASP tool is a generic tool for evaluating the strengths and weaknesses of any qualitative research methodology, i.e., risk of bias. It includes ten questions that help assess the appropriateness of the methods used, as well as the clarity and relevance of the findings [30].

The analysis of the subjective experiences was performed using the digital tool Confidence in the Evidence from Reviews of Qualitative Research (GRADE-CERQual). This structured approach evaluates the reliability of qualitative research through a systematic and transparent framework. It assesses confidence in individual review findings based on four components: (1) methodological limitations, (2) coherence, (3) adequacy, and (4) relevance. Confidence is graded as high, moderate, low, or very low. All findings were initially marked with high confidence and downgraded if important concerns arose about any GRADE-CERQual components. Each review was examined independently by two reviewers (LM and TM), and their assessments were then discussed to reach a consensus on eligibility. The author EA was consulted to verify the final assessments.

### Data collection

A data extraction form was designed, developed, and tested independently on five randomly selected studies by two of the authors (TM and LM) and assessed by a third author (AR) to ensure consistency in extraction. The extracted data included information on the characteristics of the included studies and study participants, such as the authors, year, country, condition (type of pain: acute, chronic, or cancer), mean age of patients, male-female ratio, participant characteristics, measurement variables, and key findings.

### Data analysis

The findings and assessments were summarized in a CERQual Evidence Profile. This profile provided a brief summary of each finding, explanations for the judgments made, the overall confidence level, and references to contributing studies. It acted as a detailed record of the analysis and reasoning behind the confidence ratings [31].

All summarized review findings were subsequently organized (by LM, TM, MC, NC, and GDC) into review-finding groups, resulting in four initial themes. These themes originated from combining data aligned with the review's goals. The themes were reviewed to ensure they accurately represented the data. Each theme was named, and its meaning was clarified. A Summary of Qualitative Findings (SoQF) Table (Table 1) was created to present the final findings, including summaries, confidence ratings, and study references [91]. Finally, key quotes were extracted from the referenced studies to support the findings within each theme. These quotations highlighted key insights and provided direct evidence to corroborate the presented statements.

**Table 1:** The extracted study characteristics of each individual article of the 62 articles analyzed in the systematic review

Author(s), year	Country	Condition (type of pain: acute, chronic or cancer)	Age of patients (mean (range))	Exposure groups (no. of patients)	Male:female ratio	Participant characteristics	Measurement variables	Key findings	Assessment of confidence
Aegler & Satink 2009 [32]	Netherlands	Chronic pain	46 (35–67)	8	3:5	Chronic musculoskeletal non-malignant pain for at least 3 years; Unable to work for at least 3 years; German speaking; Living situation: n = 4 families with children n = 3 alone n = 1 with a partner	Subjective experience of chronic pain; Occupational Performance	Patients with chronic musculoskeletal pain struggle to take breaks and resume activities, despite being motivated to start them	High confidence
Antunovich et al. 2021 [33]	New Zealand	Chronic pain	50.92 ± 14.52	48	14:34	Adults (older than 18 years); Spoke fluent English; Diagnosed with CRPS at the time of recruitment; Relationship status (n): single (11), in a relationship (4), married (29), divorced/separated (4); Employment (n): full-time (5), part-time (4), retired (11), student (2), sickness benefit (2)	Subjective experience of chronic pain	Patients with CRPS often face intense physical and emotional challenges. Leading to feelings of powerlessness, a desire to understand the condition's cause, and sometimes a mental disconnection from the affected limb to maintain a separate sense of self	High confidence
Armoogum et al. 2023 [22]	England	Cancer pain	62.4 (38–78)	19	5: 14	Cancer type (n): breast (10), head and neck (2, head and neck and Non-Hodgkins Lymphoma (1), Non-Hodgkins Lymphoma (1), endometrial (1), ovarian (1), testicular (1), Hodgkins Disease (1), multiple myeloma (1); Time since end of cancer treatment (n): < 1 years (1), 1–5 years (5), 5–10 years (4), > 10 years (9, Range 18 months—48 years; Time from end of cancer treatment to developing chronic pain (n): < 1 year (11), 1–5 years (1), 5–10 years (1), > 10 years (6)	Subjective experience of chronic/ cancer pain	Cancer survivors with chronic pain often feel unsupported, unseen and struggle to access care or information	High confidence
Blomqvist & Edberg 2002 [34]	Sweden	Chronic pain	85 (± 6.0)	90	24:66	75 years and over; being in persistent pain (defined as pain more or less daily for more than 3 months); receiving help from staff, and being able to participate in an interview; Living area (n): inner city/suburban (55), urban (35); Dwelling (n); own home (64), sheltered accommodation (26); Help with instrumental activities of daily living (n): daily (61), less often than daily (29); Help with personal activities of daily living (n): daily (27), less often than daily (63)	Subjective experience of chronic pain; impact on daily life	Chronic low back pain impacts daily life, causing sadness, helplessness, and fear of worsening symptoms. Patients hope normal activities might ease the pain	High confidence
Campbell & Cramb 2008 [35]	England	Chronic pain	(36–66)	12	3:9	Five were employed, three retired and four were unemployed because of ill health. Nine were married, three single or divorced. All were of white, British ethnic origin. Participants reported chronic pain, predominantly low back pain, ranging from 7 months to 22 years in duration	Subjective experience of chronic pain	Three main themes: dependence and social withdrawal; being ‘normal’ in comparison to others; and striving for self-management	Moderate confidence
Christensen et al. 2023 [36]	Denmark	Chronic pain	56 (19–64)	11	3:8	Individuals experience chronic musculoskeletal pain that had persistent for a period of longer than 3 months and was associated with significant emotional distress; Current job status (n): sick leave (10), student (1); Pain duration (Range: 10 months-39 years, Median = 4 years)	Subjective experience of chronic musculoskeletal pain; pain self-management challenges	Patients with chronic musculoskeletal pain struggle with identity, uncertainty, and a lack of support, but acceptance, optimism, pain management strategies, and strong emotional support help them cope	High confidence
Clarke et al. 2012 [37]	USA	Chronic pain	73 (66–89)	23	7:16	Sixteen participants were Caucasian, the remainder Chinese. All participants were retired, with the exception of one man who worked full-time, 15 lived alone and 8 lived with their spouses	Older adults subjective experience of chronic pain	Older adults describe chronic pain through stories and metaphors, finding it difficult to rate, and highlight how it impacts daily activities, making tasks harder and sometimes leading to isolation	High confidence
Crowe et al. 2010 [38]	New Zealand	Chronic pain	55.1 (25–80)	64	33:31	18 years of age or above, and suffering from chronic low back pain defined as: pain, muscle tension, or stiffness localised below the costal margin and above the inferior gluteal folds, with or without leg pain (sciatica) persisting for longer than 12 weeks	Subjective experience of living with chronic back pain	Four main themes were found: the unpredictability of pain, the need to stay alert, the detachment from the body, and changes in self-image	High confidence
Cummings et al. 2017 [39]	USA	Chronic pain	(29–78)	20	4:16	Race: White 14, Black 2, Asian 2, Native American 1, Other 1 Marital Status: Single/never married 4, Married 11, Widowed 3, Divorced 2, Education Level, High School 7, Some college 7, Bachelor’s degree 5, Graduate degree 1 Employment Status: Working 5, Unemployed/not working 7, Retired 8 Health insurance: Private 6, Medicare + private supplement 7, Medicare + Medicaid 2, Medicaid	Subjective experience of living with chronic pain	Participants described chronic back pain as a lonely struggle that limited work, free time, and social connections, with the struggle between wanting to manage pain independently and needing help from others causing anxiety	Moderate confidence
De Souza & Frank 2007 [40]	UK	Chronic pain	49.3 (SD 15.2, 27–79)	11	5:6	Marital status (n): Marries (10), Single (1) Occupation (n): Unemployed (2), Sick leave (1), Unable to work (2), Retired (2), Housewife (3), Employee (1)	Subjective experience of chronic pain	Participants highlighted struggles with sleep, mobility, and independence, focusing on losses in daily life, and suggested that helping adjust to these changes may be more beneficial than promising a pain-free life	Moderate confidence
De Souza & Frank 2011 [41]	UK	Chronic pain	49.3 (sd: 15.2, range: 27–79)	11	5:6	Marital status (n): Marries (10), Single (1) Occupation (n): Unemployed (2), Sick leave (1), Unable to work (2), Retired (2), Housewife (3), Employee (1) The mean total duration of SP was 10.4 (sd: 8.7, range:0.5–29) years; The mean episode duration of pain was 16.8 (sd:27.5, range: 1–96) months	Subjective experience of living with chronic pain	Patients worried about the impact of pain on family, regretted losing work ability, and feared financial consequences, highlighting how chronic pain affects both the individual and their relationships	Moderate confidence
Dow et al. 2012 [42]	UK	Chronic pain	48.6 (24–80)	30	13:17	Employment (*n*): Retired (11), Unemployed (9), Employee (10)	Subjective experience of living with chronic pain	Frustration from chronic pain builds over time due to its invisibility, diagnostic challenges, and unmet goals	High confidence
Duggleby 2000 [43]	USA	Cancer pain	”Over the age of 65”	11	5:6	Ethnicity (n) = Caucasian (10), African American (1) Religious preference: Protestant (10), No preference (1) Primary caregivers (*n*): Spouses (9), Daughter (1), Sister-in-law (1) Years of education (*n*): 9 (1), 11 (1), 12 (7), 15 (2) Type of cancer (*n*): Lung cancer (5), Rectal cancer (2), Uterine cancer (1), Breast cancer (1), Transitional cell (1), Brain cancer (1)	Subjective experience of living with cancer pain	Three themes: multiple sites of pain, hierarchy of pain, and strategies used to decrease pain. Patients experienced chronic, acute and physiological pain, with physiological pain being described as the worst	High confidence
El-Haddad et al. 2018 [44]	Australia	Acute pain (acute low back pain)	61.2 (36–87)	14	5:9	Previous episode of acute LBP (*n*): Yes (7), No (7) Occupation (*n*) = Retired (8), Employee (6) Country of birth (*n*) = Australia (8), Greece (1), Iraq (1), Spain (1), Serbia (1), Italy (1) Level of education (*n*) = High school (7), Tertiary (7)	Subjective experience of acute pain (low back pain)	Patients with acute low back pain experienced severe pain and had trouble explaining their condition, finding the healthcare system confusing and hoping treatment would help them return to normal, highlighting the need for clear guidelines and consistent care	High confidence
Esson et al. 2020 [14]	Canada	Chronic pain	(20–65)	12	5:7	Occupation: Employed (3), Unemployed/student (6),Retired (3)	Subjective experience of chronic pain	Four themes emerged: Invisibility, Ambivalence, Social isolation, and Stigmatization and marginalization	High confidence
Fisher et al. 2007 [45]	USA	Chronic pain	(35–87)	13	4:9	Most of the participants lived with a family member. Most were employed at the time of study Ten of the 13 participants had completed at least some college, and 7 of the participants held college degrees. The average length of time participants had experienced pain was approximately 17 years. The participants reported diverse types of pain: back or neck pain (7); cluster or migraine headaches (or both) (3); and some had pain in other areas, including the hips, extremities, and joints	Subjective experience of living with chronic pain	Chronic pain causes emotional distress, affects daily life, strengthens relationships, and sparks creative solutions as participants adapted by changing routines and finding new meaning	High confidence
Hallberg & Carlsson 2000 [46]	Sweden	Chronic pain (fibromyalgia)	43 (22–60)	22	0:22	The women’s educational background ranged between 7 and 15 years of schooling and 19/22 women were married or living together with a partner. Ten women had been hospitalized for 4–6 weeks, whereas 12 were out-clinic patients of the Chronic Pain Unit. Except for hospitalization, these women met the same inclusion criteria as the remaining sample	Subjective experience of living with chronic pain	Chronic pain, especially in fibromyalgia, impacts daily life and can harm mental and social well-being. It’s important psychological support helping to manage pain and adopt healthier habits	High confidence
Hassankhani et al. 2023 [19]	Iran	Cancer pain	39.4 ± 7.73 years (21–55)	17	4:13	Above the age of 18, being aware of their cancer diagnosis, experienced and lived with cancer-related pain, receiving treatment with chemotherapy, radiation, or surgery, and understanding and speaking Persian or Turkish. Marita status (*n*) = Married (12) Education level (*n*) = Illiterate (2), Primary school (3), High School (3), University (8) Religion (*n*) = Shia Muslim (11), Sunni Muslim (6) Clinical diagnosis (*n*) = Colon cancer (3), Vulvar cancer (1), Breast cancer (5), Gastric cancer (1), Lymphoma (3), Lung cancer (1), AML (3) Disease status, metastatic (*n*) = 13	Subjective experience of living with cancer pain	Patients often see pain as God’s will, and support from family, doctors, and nurses, along with better pain management and social support, helps with coping	High confidence
Hellerstedt-Börjesson et al. 2016 [47]	Sweden	Cancer pain	(30–79)	15	0:15	Marital status: Living alone 1, Living together with a spouse/partner/children 14 Children: At home 5, Grown-up children 10 Education: Basic level 4, High school 10, University 1 Employment status: Currently working 12, Retired 3 Treatment with chemotherapy in doses of 75 mg/m^2^ or more of epirubicin and docetaxel, respectively. Scoring pain of 4 or greater on a visual analog scale (VAS)	Subjective experience of cancer pain	Cancer patients' experiences with chemotherapy pain include past physical changes and isolation, present pain affecting daily life, and future worries about continued pain and vulnerability	High confidence
Hervik et al. 2023 [48]	Norway	Chronic pain (chronic headaches)	51.2 (38–68)	16	4:12	One participant had completed basic education (primary school), four had completed secondary school and eleven had completed higher education. Only three participants worked full-time, the majority (*n* = 7) worked part- time and three of the participants could not work at all. The reduction in capacity to work was due to headache symptoms in all 10 cases, they all received some degree of disability allowance. Two participants were retired, and one was on maternity leave	Subjective experience of chronic pain	Pain impacted participants' work, identity, relationships, and social life, with trauma and life events triggering headaches, while shame and stigma worsened chronic pain and reduced their sense of fulfillment	High confidence
Horment-Lara et al. 2022 [49]	Chile	Chronic pain	66.2 (43–76)	10	0:10	Occupation (*n*) = Homemaker (8), Housewife (1), Cook (1)	Subjective experience of chronic pain	Participants had negative beliefs about pain, which made them expect a poor recovery and avoid activities. These beliefs were influenced by healthcare advice. Despite their beliefs, the women still felt capable of managing their situation	High confidence
Hovind et al. 2013 [50]	Norway	Cancer pain	55 (44–65)	8	0:8	Women diagnosed with early-stage breast cancer with- out metastasis Chronic pain rated at 3–5 on a 11-point numerical rating scale (NRS), age between 18–65 years, Able to understand spoken and written Norwegian. Marital status (*n*) = Married/cohabiting 6, Divorced 1, Single 1 Education (*n*) = Primary school/comprehensive school 2, Junior college 1, Postgraduate college 3, University 2 Work (*n*) = Full-time 3, Part-time because of diagnosis (80% and 50%) 2, Temporary disabilities 1, Disabilities prior to diagnosis 2 Adjuvant treatment (*N*) = Radiotherapy 7, Chemotherapy 4	Subjective experience of cancer pain	The women expected pain after surgery, but didn’t expect it to become chronic. They weren't given much information about pain, took few painkillers, and were wary of medication due to past reactions to drugs. They received little support for their chronic pain, but most were still active and worked outside the	Moderate confidence
Hylén et al. 2020 [51]	Sweden	Acute pain	52 (41–80)	16	8:8	Diagnosis (*n*) = Surgical (11), Medical (5), Received analgesics (*n*) = 16 Intubated (*n*) = 9 Non-intubated = 7 CAM ICU before interview (*n*) = Negative (16) Previous ICU experience (*n*) = 0 Length of stay in ICU: range in days (md) = 15–22	Subjective experience of acute pain; pain assessment	Participants described their pain as intense and isolating, but good care, pain management, and clear communication helped them feel more in control	High confidence
Igwesi-Chidobe et al. 2017 [52]	Nigeria	Chronic pain (chronic low back pain)	(30–69)	30	15:15	Main occupation (n) = Manual workers (14), Non-manual workers (3), Traders (7), Civil servants/retired civil servants (6) Religion (Christian denomination) (*n*) = Protestant Pentecostal (18), Catholic (8), Methodist (3), Anglican (1) Marital status (*n*) = Married (26), Widowed (3), Single (1) Educational level completed (*n*) = Primary (11), Tertiary (7), Secondary (6), None (6) Literacy (ability to read and write) (*n*) = Illiterate (inability to read and write) (13), English (9, English and Igbo (8) Comorbid conditions (*n*) = Hypertension (4), Diabetes (1), Knee osteoarthritis (1)	Subjective experience of living with chronic pain	Beliefs about back pain, shaped by factors like work, culture, and location, influenced coping, with positive beliefs encouraging healthy coping and negative ones causing frustration with healthcare	High confidence
Ilgunas et al. 2023 [53]	Sweden	Chronic pain (temporomandibular disorders)	31.5 (20–65)	16	6:10	Individuals aged 18 years or older who reported frequent orofacial pain or jaw catching/locking at their latest dental check-up at the PDHS	Subjective experince of chronic pain	Participants with TMD sought dental care only when symptoms worsened, but faced limited access and dissatisfaction with treatment. Many felt ignored by providers and wanted better communication and involvement in decisions about their care	High confidence
Jerlock et al. 2005 [54]	Sweden	Acute pain (chest pain)	51 for women and 37 for men (18–63)	19	11:8	Six informants were manual workers, four were owners of a business, three held leadership positions, two were receptionists, one was a musician and one was a student. Two informants were receiving disability pensions and four were on temporary sick leave. Three women and two men were immigrants	Subjective experience of acute pain	Unexplained chest pain significantly disrupts daily life, causing fear, anxiety, uncertainty, stress, and weakness. Patients often seek help when they are unsure of the pain's cause or when symptoms worsen	Moderate confidence
Jones et al. 2023 [55]	USA	Cancer pain	58.8 SD = 7.1 (48–73)	13	2:11	Education (*n*) = HS or partial college or associates degree (4), Bachelor’s degree (4), Graduate degree (5) Financial status (*n*) = Comfortable (5), Have enough to make ends meet (7, Do not have enough (1) Partnered status (*n*) = Single (1), Married (8), Divorced (4) Employment (*n*) = Employed (6), Unemployed (1), Retired (2) Sick leave (3), Volunteer (1) Cancer type (*n*) = Breast (6), Head and neck (5), Lung (2), Sarcoma (1) Time since treatment (*n*) = 3 Months to 1 year (3), Between 1 and 3 years (3), Between 3 and 6 years (2), Between 7 and 10 years (4), More than 11 years (1) Pain interference (mean, SD) 4.3 ± 1.6 Pain severity (mean, SD) 4.4 ± 1.6 No. of sites of pain (mean, SD) 11.5 ± 6.3	Subjective experience of cancer related chronic pain	Cancer survivors with chronic pain felt misunderstood and isolated, facing stigma over opioid use and lacking support, which led them to manage pain through trial and error	High confidence
Juuso et al. 2011 [56]	Sweden	Chronic pain	54 (38–64)	15	0:15	Nine women were married, two were co-habiting, and four were single. All had children and five had minors still living at home. Eleven women had a vocational education, three had elementary schooling, and one had a university education. Four women were employed or looking for work, six were on sick leave, three received a state pension, and two a disability pension	Subjective experience of chronic pain	The findings show that women with fibromyalgia face two main struggles: dealing with constant, unpredictable pain and being doubted by others because their pain isn't visible	Moderate confidence
Juuso et al. 2016 [57]	Sweden	Chronic pain (fibromyalgia)	54 (38–64)	15	0:15	Marital status (*n*) = Single (4), Married (9), Cohabiting (2) Children (*n*) = Yes (15) Education (*n*) = Vocational (11), University (1), Elementary (3) Employment (*n*) = Sick leave (6), Disability pension (2), Employed (3), Unemployed (1), State pension (3) Year since diagnosis = 1–20 years Duration of symptoms = 6–37 years	Subjective experience of living with chronic pain (fibromyalgia)	Women with fibromyalgia often felt a sense of loss and fear about their future work prospects, as they struggled to accept changes in their ability to work and the impact on their careers	High confidence
Kurz & Hebron 2024 [58]	UK	Chronic pain	57.8 (50–69)	6	2:4	Received multiple treatments in the past. In addition, two had received injections and one person had undergone surgery. Employment (n) = Retired (2), Full-time (3), Part-time (1)	Subjective experience of chronic pain	Participants experienced a loss of self due to pain, later finding a new "normal" through acceptance and trusting their instincts. Anxiety and fear persisted but became more manageable, highlighting the need for more attention to society's role in coping	High confidence
Kwon & Kim 2021 [59]	Korean	Chronic pain	41.1 (20–65)	11	6:5	Marital status (*n*) = Married (8), Unmarried (3) Diagnosed with CRPS Cause of diagnosis (*n*) = Road traffic accident (6), Fracture (1), Military training (1), Intravenous injection (1), Surgery in 1988 (1), Neurosurgery (1)	Subjective experience of chronic pain	Pain was hard for patients, and lack of support made it worse. Offering better support and education for families and caregivers could help improve care	High confidence
Larsson & Wijk 2007 [60]	Sweden	Cancer pain	70 (54–82)	3	2:1	Different types of cancer, experiencing both nociceptive and neuropathic pain in 2003. The patients were under palliative inpatient or outpatient care	Subjective experience of cancer pain	Cancer patients feel intense pain and worry about the future, sometimes hiding or sharing their pain. With good pain management and support, they can regain control and find new purpose in life	Moderate confidence
Law et al. 2019 [61]	China	Chronic pain	(64.3 (65–80))	20	4:16	Patients experiencing pain (chronic/acute or cancer-related) and their strategies for handling pain	Subjective experience of living with chronic musculoskeletal pain (CMSP) with multimorbidity (MM)	CMSP impacted elderly patients with MM on both a physical and psychosocial level. The study also lifts “the barriers to pain care in the community, and the perception and strategies on pain management”	High confidence
Lundberg et al. 2007 [62]	Sweden	Chronic pain	51 (30–64)	10	5:5	Patients with heterogeneous persistent nonmalignant musculoskeletal pain(duration > 6 months)	Subjective experience of chronic pain; “moving with pain”	Patients suffering from heterogeneous persistent nonmalignant musculoskeletal pain resulted in failed adaptation, identity restoration, and finding a way out	High confidence
Mackichan et al. 2013 [63]	England	Chronic pain	(67–92)	31	15:16	Patients experiencing chronic pain (defined as pain that was experienced either all the time or intermittently for at least 3 months)	Older adults subjective experience of chronic pain	Patients altered or reduced their social and physical activities to prevent pain exacerbation	High confidence
Mannerkorpi et al. 1999 [64]	Sweden	Chronic pain	(24–54)	11	0:11	Patients fulfilling the ACR criteria for FMS. Three patients worked full-time, one worked part-time and studied part-time, three worked part-time and received gov-ernment health insurance benefits for sick leave or a disability pension for the remaining time. Two received their benefits from rehabilitation programmes, one studying part-time and the other participating in a part-time work trial. Two respon-dents were on full-time sickness leave or disability pension	Subjective experience of chronic pain; quality of life, living with FM	Four main themes were identified for patients suffering from FM; Struggling, Adapting, In despair and Giving up	High confidence
Martin 1989 [65]	USA	Chronic pain	(45.5 (27–70))	15	9:6	Individuals with low back pain	Subjective experience of chronic pain	Patients expressed that the chronic pain they experienced affected every aspect of an individual's life	High confidence
Martin et al. 2015 [66]	Brazil	Acute pain	(44.5 (18–71))	29	13:16	In the majority of cases, the trauma was caused by domestic accidents and could be classified as minor or moderate	Subjective experience of acute pain and its consequences	The acute pain from minor trauma was influenced from different factors such as sociocultural, spiritual, biological and emotional	High confidence
McHugh & Thoms 2001 [67]	England	Chronic pain	(23–86)	245	103:151	Employed, unemployed, retired, homemaker, in education or medically disabled	Subjective experience of chronic pain	Patients that suffered from chronic pain experienced that the pain affected their job and their daily tasks. One third of the participants identified themselves as medically disabled	Moderate confidence
Michaëlis et al. 2015 [68]	Denmark	Chronic pain	(33–63)	13	0:13	Married, divorced, between 0 and 6 children	Subjective experience of chronic pain; quality of life, coping with the pain	The experience of chronic pain led to consequences for women’s lives, as their daily living was limited by the physical impact of the pain. Various pain management strategies were mentioned by the participants	High confidence
Nielsen et al. 2013 [69]	Australia	Chronic pain	(50 (30–70))	20	7:13	Different education levels, Full-time employment, Part-time employment, Disability Support, Pensioner, Retired, Student, Married/marriage-like, relationship, Divorced, Widowed, Single	The subjective experience of living with chronic pain	According to the study, chronic pain treatment is often affected by poor communication and lack of coordinated care by the health providers, which patients cannot control	High confidence
Nilsen & Anderssen 2014 [70]	Norway	Chronic pain	(42.7(26–63))	20	10:10	The patients suffered from different types of diseases or injuries (e.g. neck and back pain, traffic injuries)	Subjective experience of living with chronic pain	Patients suffering from long-term chronic pain often manage their pain on their own, because of lack of long term support. Patients also feel isolated in managing their pain. Finding a job adapted to their state was a main tactic for many participants	Moderate confidence
Ojala et al. 2015 [71]	Finland	Chronic pain	(48 (26–73))	34	15:19	Half of the participants were retired,and a fifth worked full-time. Each of the participants could walk without assistance but many needed help in tasks including household work. Most of the participants used a combinationof medications	Subjective experience of living with chronic pain, VAS	Patients struggling with chronic pain expressed that it was more challenging with the psychosocial consequences rather than the physical pain itself. Such as distress, loneliness, lost identity, and low quality of life which were their main problems	High confidence
Orujlu et al. 2022 [72]	Iran	Cancer pain	40.2	14	6:8	Working, housewife, married, different education levels, Colon cancer, Breast cancer, Gastric cancer, Lymphoma, Lung cancer and AML	Subjective experience of living with cancer pain, NRS	Obstacles such as negative attitude, limited knowledge and the influence of religious beliefs were some of the barriers to effective cancer pain management	High confidence
Paulson et al. 2002 [73, 74]	Sweden	Chronic pain	(47 (41–56))	14	14:0	Two participants worked full-time but in different positions than before, 5 worked part-time, 2 were on leave of absence due to illness, 1 was on paternity leave, 3 received disability pen-sions, and 1 man was temporarily unemployed on a voluntary basis. Symptoms for between 4 and 24 years	Subjective experience of chronic pain	Men experiencing chronic pain, fibromyalgia, experience changes in their body, sense of self, and relationships. Patients were required to find balance during different phases of the condition, as they tried to maintain a tolerable existence	Moderate confidence
Paulson et al. 2002 [73, 74]	Sweden	Chronic pain	(41–56)	19	19:0	Married,or live together with a female partner	Mens subjective experience of living with chronic pain	Themes emerged from the study were; Feeling afraid of being looked upon as being a whiner, Feeling like a guinea pig, and Feeling hopeful	Moderate confidence
Robinson & Maree 2024 [75]	South Africa	Cancer pain	(52.2 (20–76))	20	9:11	Patients had to be 18 years and older, diagnosed with cancer	Subjective experience of people living with cancer	Patients diagnosed with cancer experience total pain and emotional pain can outweigh physical pain. Patients experienced a lack of communication about the cancer and its treatment	Moderate confidence
Sakyi et al. 2024 [76]	Ghana	Acute pain	20–40	9	3:6	Patients who have undergone surgery and experienced post-operative pain	Subjective experience of acute pain	Participants suffering from acute pain after undergoing surgery have different pain expectations and experiences. the pain affected their activities of daily living and emotions. Coping strategies: Support from nurses and or personal strategies	High confidence
Sallinen & Mengshoel 2019 [77]	Norway and Finland	Chronic pain	(43 (24–51))	6	6:0	Men diagnosed with fibromyalgia and had symptoms for at least two years before receiving the diagnosis. One of the participants was on permanent disability pension due to fibromyalgia, one was doing reduced working hours (30 h/week), and three were working full-time	Subjective experience of living with chronic pain	Men experiencing chronic pain (FM) expressed how the pain affected their social relations and family life. It was common with changed activities in order to not worsen/aggravate the pain	High confidence
Simonsen-Rehn et al. 2000 [78]	Finland	Cancer pain	28–70	10	2:17	Patients diagnosed with cancer	Subjective experience of living with cancer pain, pain management	Patients expressed how healthcare providers often provide inadequate pain relief and fail to address their pain. This, along with the cancer, lead to emotions that change the patient's sense of balance, increasing vulnerability	Moderate confidence
Sturge-Jacobs 2002 [79]	Canada	Chronic pain	20–57	9	0:9	Patients diagnosed with FM for at least 1 year;	Women's subjective experience of living with chronic pain (FM), QoL	Female patients suffering from FM experienced constant pain, fatigue and sleeplessness as a result of the pain. Further result from living with the pain was customizing their lives, lack of comprehension from friends and family members	Moderate confidence
Söderberg et al. 1999 [80]	Sweden	Chronic pain	(43.3(35–50))	14	0:14	Women who has experienced FM symptoms for about 1 to 25 years, and time since diagnosis ranged between 0.5 and 6 years. Nine participants had trade/upper secondary school education, 3 compulsory education, and 2 university/ higher education. Most of the participants were married, 3 were divorced, 1 was a common-law wife, and 1 was single. Nine of the participants were employed part time, 2 full time, and 3 were not working	Women's subjective experience of living with chronic pain (FM), QoL	FM affected the female patients' lives in various ways. The emerging themes from the study were: “loss of freedom, threat to integrity and a struggle to achieve relief and understanding”	Moderate confidence
Walker et al. 1999 [81]	England	Chronic pain	28–80	20	12:8	Patients diagnosed with chronic low back pain,	Subjective experience of chronic pain	The patintens experienced that the pain dominated their daily life. The key findings were categorized into: “The pain takes over, Sense of loss, In the system, They don’t understand and coming to terms”	Moderate confidence
Webber et al. 2011 [82]	England	Cancer pain	(59.2 (32–76))	10	5:5	Patients with diagnosis of cancer	Subjective experience of living with cancer, breakout pain	The breakthrough pain experienced by the patients impacted their daily lives and led to patients making adjustments in their life to reduce the pain	High confidence
Westergården et al. 2021 [83]	Sweden	Chronic pain	45–67	19	0:19	Women with chronic widespread pain (CWP), 13 patients working full or part-time, 3 on sick leave and 3 retired	Women's subjective experience of living with chronic pain	Patients living with CWP experience that they either live life managing the pain or are being overwhelmed by the pain. They experience challenges such as “feeling unseen, struggling with limitations, and adapting daily to varying pain levels”	High confidence
Wolf 2006 [84]	Sweden	Chronic pain	(55.3 (21–77))	14	3:11	Patients suffering from chronic pain between 5 months—20 years	Subjective experience of chronic pain, VAS,	Patients experienced distrust from healthcare workers, had no support for pain management, and different medication was used for ongoing pain	High confidence
Wolf et al. 2008 [85]	Sweden	Chronic pain	(55.3 (21–77))	14	3:11	Seven of the patients were married, 2 widowed, and 5 single. Of the 10 patients who had children. Six patients were employees or self employed, 5 were retired, 2 were sick-listed, and 1 patient was a student. Pain duration ranged from 3 months to 20 years	Subjective experience of living with chronic pain	Patients expressed that the pain was difficult to communicate. They experience feelings of hopelessness, resignation and lack of faith	High confidence
Wolf et al. 2016 [86]	Sweden	Chronic pain	(55.3 (21–77))	14	3:11	Patients with no reasonable explanation for the chronic oro- facial pain condition or a pain behaviour that, to an experienced clinician, appeared to be incongruent with the pain described	Subjective experience of living with non specific orofacial pain	Living with nonspecific chronic orofacial pain led to negative emotions dominating their day as well as a fear of personal weakness	High confidence
Xia et al. 2024 [87]	China	Cancer pain	18–> 60	17	10:7	Patients pathologically diagnosed with malignant tumor in clinical stage III or stage IV; that showed symptoms of distant metastasis. Hospitalized with recurrent disease and pain lasting for over three months. A self-reported pain intensity > 3 based on an 11-point Numerical Rating Scale (NRS); and were aware of their own conditions	Subjective experience of living with cancer pain, NRS	Pain acceptance in advanced cancer patients involved four themes: “pain catastrophizing, rumination, avoidance coping, and constructive action”	High confidence
Xu et al. 2019 [88]	China	Cancer pain	(55.9 (37–75)	12	6:6	66.7% were graduates of high school or below, 83.3% were married, and 50% were farmers. Four patients had lung cancer, three had breast cancer, two had colon and rectum cancer, and the others had myeloma, liposarcoma, and non- Hodgkin’s lymphoma	Subjective experience of living with cancer pain, NRS	Cancer patients experienced that the pain had an impact on daily life and work; patients had different coping strategies (medication, behavioral, social, and spiritual support)	High confidence
Yeowell et al. 2021 [89]	England	Chronic pain	(54 (30–70))	9	8:1	Patients diagnosed with Osteoarthritis (OA)	Subjective experience of living with chronic pain	Patietns experiencing OA expressed how the pain affected their daily life and led to seizing of physical activities because of the fear of pain. The pain impacted their self-identity and led to a loss of self-worth	High confidence
Yildizeli Topcu 2018 [90]	Turkey	Chronic pain	(49.54 (18–82))	86	32:54	Patients with chronic joint pain	Subjective experience of living with chronic pain	The study states that there is a correlation between patients perception of pain, mental health and wellbeing	Moderate confidence

## Results

### Literature search outcome

**Fig. 1:**
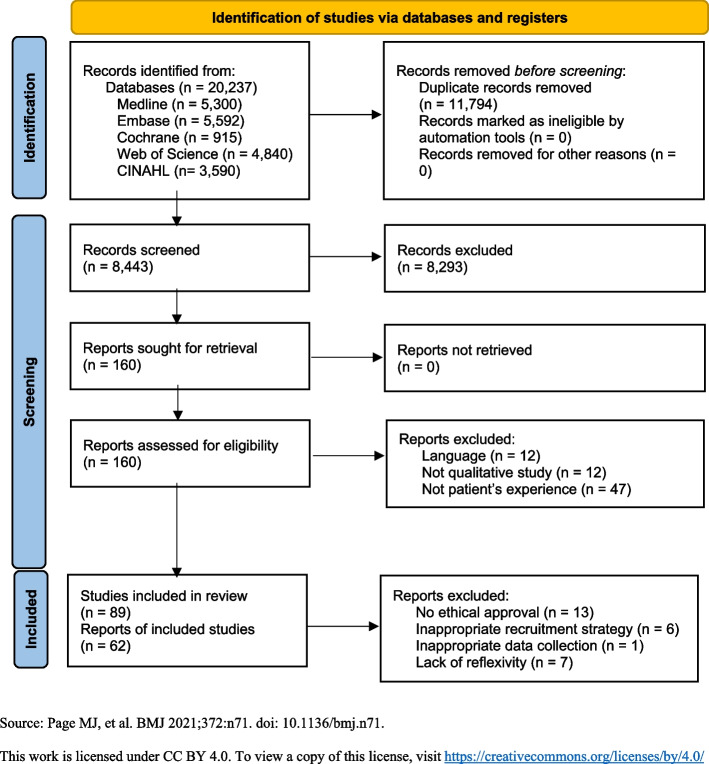
PRISMA 2020 flow diagram showing the identification and inclusion process of the 62 studies included in this qualitative systematic review

The full digital literature search resulted in 20,237 studies from all included databases. After the removal of 11,794 duplicates, a total of 8,443 articles were screened by title and abstract. The screening resulted in 160 full-text articles that were sought for retrieval, of which all of them were retrieved. Then, 71 full-text articles did not meet the inclusion criteria, thus resulting in 89 included studies. The full process of inclusion is shown in the PRISMA flow diagram (Fig. 1).

### Methodological assessment

The 89 articles were assessed as either having “no/very minor,” “minor,” “moderate,” or “serious” methodological limitations. An open discussion and consensus were reached between the three authors, TM, LM, and MC, about the assessment of articles. The following categories were reasons for articles to be assessed as “moderate” or “serious” methodological limitations: (a) lack of ethical approval from an ethics committee; (b) lack of reflexivity by the authors; (c) lack of rigor in data presentation and/or insufficient selection of participants; (d) or the lack of disclosure of the selection method. The individual assessment of the 89 articles is presented in Table S3.

### Characteristics and confidence assessment of included studies

Based on the confidence assessment of the 89 included articles, 62 showed moderate (*n* = 18) [35, 39–41, 50, 54, 56, 60, 67, 70, 73–75, 78–81, 90] to high (*n* = 44) [14, 19, 22, 32–34, 36–38, 42–49, 51–53, 55, 57–59, 61–66, 68, 69, 71, 72, 76, 77, 82–89] confidence, thus assessed as having high enough quality suitable for this review. The primary reason for the exclusion of articles was the lack of ethical approval by an ethics committee, with 13 articles being excluded [92–104]. Lack of rigor in data presentation was also a contradictory factor to an article being evaluated as low or very low quality, resulting in the exclusion of 6 articles [105–110]. Additional reasons for 7 articles evaluated as low or very low quality were an insufficient selection of participants [18, 111–115] or the lack of disclosure of the selection method [116]. One article was also excluded because it did not focus on the subjective experience and was therefore deemed irrelevant [117], shown in Fig. 1.

The studies included were interview studies investigating the subjective experiences of adults (> 18 years old) with acute, chronic, or cancer pain. The characteristics of the included studies are presented in Table 1, including information on the following: (a) Author(s), year of publication; (b) Country; (c) Condition (i.e., type of pain: acute, chronic, or cancer); (d) Age of patients (mean (range)); (e) Exposure groups (no. of patients); f) Male-to-female ratio; (g) Participant characteristics; (h) Measurement variables; (i) Key findings; and (j) Assessment of confidence.

### Summary of review findings

The review findings summarized in the CERQual Evidence Profile resulted in the following four main themes: (1) impact of pain on social life, work life, and family life; (2) challenges in healthcare access; (3) psychological impact and emotional struggles from pain; and (4) barriers to effective pain management, shown in Table 2. The reasoning, methodological limitations, coherence, adequacy, relevance, confidence of evidence, as well as reference to the studies contributing to each theme are also presented in Table 2. Below follows a presentation of each theme including key quotes extracted from the referenced studies highlighting the key insights.

**Table 2:** The summary of the review findings is presented in the four themes

Summary of review findings	Studies contributing to the review finding	Methodological limitations	Coherence	Adequacy	Relevance	*CERQual assessment of confidence in the evidence*	Explanation of CERQual assessment
Patients suffering from chronic-, acute- or cancer pain face challenges related to their social life leading to a feeling of helplessness and vulnerability, withdrawing or completely ceasing from social activities because of pain or fear of pain. The pain also contributed to a change in their work life and family- and friends dynamic as their dependability on them increased	1, 4, 12, 14, 18, 21, 22, 25, 30, 31, 40, 42, 44, 45, 49, 51, 53, 54, 56, 67, 68, 70, 71, 72, 74, 76, 81, 85, 91, 92, 102, 107, 108, 111, 114, 115, 116	Minor methodological limitations (24 studies with no/very minor methodological limitations, 11 studies with minor methodological limitations (interview questions were not provided, reflexivity not considered), 2 with moderate methodological limitations (consent not obtained, no reflexivity)	No/very minor concerns about coherence	Very minor concerns about adequacy (36 studies in total provide satisfactory rich data and one with lack of extensive analysis of the data.)	No/ Very minor concerns about relevance (studies of patients suffering from chronic, acute or cancer pain with a wide range of participants and different aspects on their issues)	High confidence	Very minor concerns regarding methodological limitations, relevance, coherence and adequacy
Patients experience challenges in accessing healthcare for their suffering of chronic, acute or cancer pain. Many report inadequate pain assessment as well as the feeling of being misunderstood, unsupported and their pain being overlooked. Overall, patients express frustration and dissatisfaction with health care	4, 10, 12, 23, 28, 49, 51, 54, 56, 72, 76, 79, 81, 86, 90, 95, 101, 102, 106, 107, 110	Minor methodological limitations (12 studies with no/very minor methodological limitations, 8 studies with minor methodological limitations (interview questions were not provided, reflexivity not considered, lack of consideration of bias), 1 with moderate methodological limitations (consent not obtained, no reflexivity)	No/very minor concerns about coherence	No /very minor concerns about adequacy (21 studies in total provide satisfactory rich data)	Very minor concerns about relevance (one study describes patients experience after attending pain services, remaining 20 studies of patients suffering from chronic, acute or cancer pain with a wide range of participants and different aspects on their issues)	Moderate confidence	Moderate methodological limitations and moderate concerns regarding adequacy of data
Patients suffering from chronic, acute, or cancer pain often struggle to manage their pain due to poor assessments, limited treatment options, and hesitation from doctors to prescribe strong medications. With a lack of appropriate information, patients often take matters into their own hands regarding pain management	6, 25, 38, 40, 44, 46, 54, 55, 61, 62, 64, 70, 72, 81, 83, 90, 101, 107, 114, 115	No/Very minor methodological limitations (12 with non/very minor and 6 studies with minor (interview questions not provided, reflexivity not thoroughly discussed) and 2 studies with moderate methodological limitations (no reflexivity, consent not obtained)	No/very minor concerns about coherence	No/very minor concerns about adequacy (20 studies in total provide satisfactory rich data)	No/ Very minor concerns about relevance (studies of patients suffering from chronic, acute or cancer pain with a wide range of participants and different aspects on their issues)	High confidence	Very minor concerns regarding methodological limitations, relevance, coherence and adequacy
Patients experiencing pain (chronic/acute or cancer -related) struggle with their mental health, often leading to psychological distress such as depression, anxiety, and stress. As they struggle to cope, patients develop tendencies towards pain catastrophizing, lower self-esteem and a feeling of loss of identity	2, 6, 10, 17, 25, 38, 40, 44, 45, 49, 55, 59, 61, 62, 70, 71, 72, 76, 81, 82, 90, 101, 102, 111, 112, 114, 115, 116, 118	No/Very minor methodological limitations (18 with non/very minor and 10 studies with minor (interview questions not provided) and one study with moderate methodological limitations (no reflexivity, consent not obtained)	No/very minor concerns about coherence	No /very minor concerns about adequacy (29 studies in total provide satisfactory rich data)	No/ Very minor concerns about relevance (studies of patients suffering from chronic, acute or cancer pain with a wide range of participants and different aspects on their issues)	High confidence	Very minor concerns regarding methodological limitations, relevance, coherence and adequacy

#### Impact of pain on social, work, and family life

The first theme identified was the “impact of chronic, acute, and cancer pain on social, work, and family life”. Patients suffering from chronic and cancer pain reported experiencing higher levels of social isolation compared to those with acute pain. In many studies, participants with chronic non-malignant and malignant pain mentioned altering plans or avoiding certain activities when pain was anticipated or present:

You know when [the pain is] going to come, and what you have to do is plan your life around it. So, in other words, usually I don’t try to plan anything [at] those times so that I know that I will hopefully be safe [45].

Oh well I can’t walk, I can’t go shopping in malls and things like that. I'm no use to my friends because they're all active, everybody wants to go for a walk and I can't [37].

Previously, when people invited me to parties or to meet people, I have abstained due to the pain . . . //So now people are tired of this, so I’m not now invited anywhere, except by the family. I’ve lost a lot of friends and social . . . //I didn’t think about it before when the pain was at its worst. I don’t know if I’ve done something wrong in these situations [83]

Acute pain also affected patients’ daily activities, but the pain appeared to have a more temporary impact on their lives:

Yes you know because of this pain hmmm I could not do things like bathing, lifting, sleeping, it was not very easy for me at all people had to even assist me in bathing and the others....[76]

Social withdrawal, in turn, affects both intimate and family relationships. According to the studies included in this systematic review, patients suffering from chronic and cancer pain experience feelings of helplessness, dependence on others, and difficulty reshaping their relationships:

...I just can’t get used to it. It is hard to accept. It is worse than the [physical] pain, I can tell you that, it is worse than [physical] pain...I can endure [physical] pain more than I can endure being helpless...Like I say, the things I ask for, I feel like they are not really unreasonable, should be expected. It is so hard to live with somebody [43].

It [chronic pain after cancer treatment] has totally, utterly ruined my life and my family’s and my marriage. Everything. . .. I mean my relationship with my husband has I would say virtually broken down. . .. It’s [chronic pain after cancer treatment] destroyed a person. It’s destroyed a person’s marriage. It’s destroyed a person’s family. It’s destroyed a person’s friendship group [22]

I feel like I would be a burden to them. I am not able to participate in all the things that we could do in the past. Water skiing and stuff like that. We were very active in the group of friends I was a part of. So, I will just become a limitation to them. If I am there, I know they will say no to some of the things they would like to do. Why should it affect them, that I have been injured? I know it's wrong, because they don’t think that way, but I just feel that it is best for them [36].

Acute pain also leads to feelings of helplessness and desperation in the moment:

[...] when I got hurt I was at home, alone, the pain came on so quickly and there was no one there to help me, it was horrible and terrifying. You need help and you don’t know who to call, you don’t know how to fix it, the pain doesn’t allow you to think, and that is what put me into a state of desperation [...] [66]

But hoping for a better day tomorrow gave the participants strength to endure their acute pain at the time:

So I had [a feeling of] hope the whole time.... I could see... and they also told me, “Look you can do this today, [which] you could not [do] yesterday” and this also gives you hope for the next day, so you think, “I just need to have this pain now ... maybe a little less pain tomorrow”... so that also helped you through the sickness and staying awake [51].

Participants with chronic cancer-related pain experienced a different kind of suffering compared to the acute pain during their cancer treatment, as it led to hidden distress. This ongoing pain impacted their self-perception:

I’m grateful. I’m blessed that I was able to fight it, and I should be happy. should be dancing on air, but it’s just not like that, and pain is one of the things added to the mix [55]

Sometimes you feel a little lesser than everybody else, cause they’re not walking around in pain and I am. No one, no one really understands they don’t understand how bad it is and that hurts [55]

A common characteristic among participants across all studies suffering from chronic and cancer pain was their experience of being on full or partial sick leave due to long-term pain. This was viewed as a temporary, uncomfortable, and undesirable situation. Many expressed a strong desire to return to work as a means of overcoming their current circumstances:

Firstly, I cannot work because of my pain, it affects my financial circumstances, and it affects me socially. It is like a line, you need to have all in succession, when you have a good economic situation, then you will also have it well socially. When you are healthy, then you are socially well too; it is a circle—when you are losing a ring, then you are losing it all. In my situation, many rings are missing, both money- and health wise. Then you just feel empty inside [68].

You come to a point when you have to stop [working] because you’re totally exhausted and have to be on sick leave more and more often. It isn’t worth it [57]

It has had a huge impact on my work. I can’t continue my original work anymore [88].

#### Challenges in healthcare access

The second major theme identified was challenges in healthcare access. One of the main factors influencing patients’ experience of healthcare was communication. Patients suffering from acute pain in intensive care felt isolated and powerless when unable to communicate their pain effectively. The term “intensive care” specifically refers to acute pain experienced by patients admitted to intensive care units, where complex clinical conditions, invasive procedures, and treatments further complicate pain management [118].

I was upset because I thought, here I am, I don’t lay down for nothing, I never have, I have got terrible pain ... He [treating doctor] said he wants me to go home ... I could have punched him you know [44].

... you’re in pain but you are being told by your doctors ‘‘No, no you haven’t got pain, it’s just a pain you’re feeling in your head’’. It just destroys you completely and it gives you a double burden to carry and that’s what had happened to me and I was destroyed by it [42]

In contrast, patients who felt supported stated that they could communicate their pain level using a familiar instrument (NRS) and had attentive caregivers who stayed close while the pain eased. Caregivers’ acknowledgement and willingness to communicate played a crucial role in restoring a sense of control:

People like this... they do their job and were professional... but there was joy and happiness and they told me jokes and it felt so enjoyable even through all of the pain... they lifted me up...and... yeah.. I could not believe it ... they see all this every day and do this all the time ... it was like they were pumping life in my body... I felt like I needed to live [51].

But we have to know the scales. We need to speak the same language....something I haven't had until this time – same language as they speak here (at the hospital) [51].

Patients suffering from chronic and cancer pain often felt that they lacked understanding of the cause of their pain:

It is just as important to understand your pain, as it is to accept it. If just I could get a proper explanation from a healthcare professional about why I am in pain, why it is the way it is, and why nothing can be done to make it better. Then hopefully all my speculations and worries would stop, so I can start concentrating my energy on some of the other challenges I need to tackle. That would make my life a lot easier. My head is completely filled with speculations about my pain and worries about what is going to happen in the future [36].

I just desperately wanted to know what was wrong with me and I mean I remember doctors saying ‘‘Oh it’s really good news. We didn’t find anything.’’ And me being really frustrated and saying ‘‘How is that good news?’’ and they were you know ‘‘You should be really pleased. There’s nothing dangerously wrong with you.’’ But I was just desperate to know what was going on because it was having such an impact on my life [42].

The lack of trust and support from healthcare professionals made participants feel misunderstood, preventing them from getting the help they needed to manage their condition:

The lack of help and responsiveness from healthcare professionals has left me with a feeling of helplessness. (..) So, I’m quite angry at them, and I’m having a hard time trusting healthcare professionals, because I thought they were there to help me. When you experience that isn’t how it is, then you become distrustful. And you start feeling that you’re not able to get the help that you need. It’s ridiculous they won’t help a young person like me. Maybe things had not come this far, if I had been able to get some help – but I couldn’t [36].

Many patients with cancer also felt like they were not informed about chronic pain after cancer treatment:

They told me it would be painful for a little while. And when I was into this several years in, I told my oncologist that the pain’s still going. She says you might have it permanently. And I said, why didn’t you tell me that in the beginning? [55]

Survivors of cancer mentioned receiving less medical attention as the cause of their pain was unclear or when no solutions were found:

They basically say, ‘Right, you’ve finished your treatment. Off you go. Goodbye. . .’ it is the feeling of being discarded [22]

#### Barriers to effective pain management

The third theme regarded pain management within patients suffering from chronic, acute, or cancer pain varies and is caused by poor assessments, limited treatment options, and hesitation from doctors to prescribe strong medications. The findings of the studies presented a variation of different pain management methods used by patients depending on the type of pain they experienced.

Keeping busy with different hobbies or jobs was an effective pain management method for chronic or cancer pain patients, acting as a distraction from their pain. Others relied more heavily on their relationships with friends and family as they expressed how it helped ease the pain.

Mari (ID16) found a way of distracting herself from negative emotions and pain. I bake bread… [48]

Distracting activities included reading, watching television, doing needlework or cooking, meditation or praying, travelling, meeting friends and attending activities run by an occupational therapy auxiliary [34].

Patients stated how their belief in God and the practice of their religion/spirituality were effective coping strategies used to reduce the feelings of distress and anxiety:

I have been a believer for a long time. Even now, I call on God hundreds of times a day. I know that pain is the destiny of God. Even now, I rely only on God. I only hope God makes me better [72].

When a patient loses himself and his faith in himself, I believe, he cannot overcome problems. To overcome all of these problems (illness and pain, for example), I must help myself; I must believe in myself [19].

The feelings of being misunderstood contribute to further barriers in pain management:

Just look at me. Can you see any of my aches and pains? My God, I look great, don’t I? So how can you believe me when I say this whole body hurts all over, all of the time? [79]

If my journey that time had great doctors, the great support system, my journey would have been different because I would not have cried so much, I wouldn’t have so many sleepless nights at home [75].

#### Psychological impact and emotional struggles from pain

The fourth major theme identified was the psychological impact and emotional struggles resulting from the pain experienced by patients. The main result of the pain across all pain types was feelings of distress and anxiety:

I became isolated, withdrawn. Pain just destroyed my life took over my life. The anxiety was running parallel with the pain…[58]

Anxiety fuels pain: the things I hear, I see, I smell and can be damp down by things that are safe, pleasurable, adding value to my life…. [58]

Patients also expressed how the experienced pain resulted in thoughts of death:

When I have this pain, I often fall asleep at night hoping I will not have a silent MI. I hope I will wake up in the morning [54].

I have never thought that all of a sudden I would get a deadly disease like cancer, but now I know, and whether or not I will get to live over Christmas I don’t know [60].

A prominent emotion shared among the different studies and pain types was the loss of self-worth and shame:

My life has no value anymore. I can’t do anything. I lost my dignity as a human being [19].

I feel bad when I am in too much pain and I ask my son or spouse for help. I cannot feed my son, meet his needs, or be a good mother. I cannot fulfill responsibilities to my family. I’m still ashamed [19].

Rose (ID7), pretended to have had a fulfilling day when the family asked her what she had been doing. She said: I am ashamed of being useless, so I pretend, to protect them and retain some degree of dignity – even though it is false [48].

## Discussion

The main finding of this review was that living with acute-, chronic-, and cancer-pain presents significant challenges, impacting overall life circumstances and mental health [22, 45, 76]. Patients often face difficulties navigating the healthcare system, feeling dismissed and unacknowledged [42]. They also experience a lack of information and difficulty finding appropriate care or support, which creates barriers to effective pain management. Consequently, patients resort to self-management strategies. A patient-centered approach is crucial for improving outcomes and providing adequate support for individuals with pain.

### Impact of pain on social life, work life, and family life

Chronic, cancer, and acute pain profoundly affect various aspects of life, including social, work, family, and intimate relationships. However, the effects differ significantly based on the duration and intensity of the pain. Chronic pain often leads to social isolation due to fatigue and fear of being misunderstood [37, 45, 83]. Similarly, cancer pain causes emotional isolation due to fear of disease progression [55]. Acute pain, however, typically seems to result in only temporary social withdrawal, with individuals generally returning to normal social activities after recovery [76].

Psychological factors, such as anxiety, depression, pain catastrophizing, and fear-avoidance beliefs, play a pivotal role in shaping patients’ pain perception and disability. The fear-avoidance model illustrates how catastrophic interpretations of pain lead to avoidance behaviors, reduced activity, heightened sensitivity, and reduced quality of life [119, 120]. Coping mechanisms like cognitive-behavioral therapy, mindfulness-based practices, and acceptance strategies have been shown to foster resilience, reduce catastrophizing, and improve functioning [120, 121].

Chronic pain significantly affects work life, often reducing productivity and leading to frequent absences or job loss due to physical challenges [57, 68]. With a prevalence of approximately 20%, the economic burden of chronic pain reaches up to US$635 billion annually in medical costs and loss of productivity [10, 122, 123]. Cancer pain has an even greater impact on employment due to both physical and emotional difficulties [88]. Acute pain, on the other hand, generally causes only temporary work disruptions. Chronic pain, especially post-cancer treatment, creates lasting emotional strain, making individuals feel vulnerable and disconnected from their former selves [43]. This often causes significant disruptions in marriages, family life, and friendships, with some patients feeling like a burden to others [22, 36]. This can further lead to withdrawal from social interactions. In contrast, acute pain disrupts family routines in the short term, and individuals generally return to their normal roles and activities once they recover [51, 66]. Hence, pain frequently undermines social and occupational functioning, leading to social withdrawal, workplace absenteeism, and role disruptions. Many patients report feelings of stigma, a loss of identity, and diminished self-worth accompanying persistent pain [124, 125]. Family caregivers often experience elevated stress, social isolation, and emotional burden, highlighting the ripple effects of pain on close relationships [126].

### Challenges in healthcare access

Healthcare relationships play a critical role in pain management, regardless of pain type. Many patients with chronic pain feel misunderstood by healthcare providers, leading to frustration and isolation [36, 42]. Many individuals with chronic pain, including those experiencing pain after cancer treatment, struggle to find effective care or solutions [55]. A lack of empathy, awareness, and long wait times exacerbate these challenges, making it difficult for patients to receive effective care [36]. Cancer-related pain often continues post-treatment, leaving patients feeling abandoned [22, 55]. The failure to acknowledge pain severity contributes to emotional distress and inadequate resources for coping.

Effective patient–provider interaction, characterized by empathy, shared decision-making, and clear communication, is essential for positive pain outcomes. Literature indicates that mismatches between clinician and patient goals, as well as a lack of empathetic listening, often leave patients feeling misunderstood or dismissed [127]. Shared decision-making tools have shown promise in enhancing trust, patient satisfaction, and adherence, and in reducing opioid misuse. Implementing such tools and training clinicians in communication skills could greatly improve patient experiences and treatment effectiveness [127].

Effective communication is essential for pain management. Patients with acute pain, especially in intensive care, often feel isolated and powerless when unable to express their pain [42, 44]. However, when healthcare providers communicate effectively and show empathy, patients feel more supported and in control. The approach and help of the nurses contribute to easing their physical pain and improve their spiritual well-being [19]. Tools like the NRS help standardize communication, ensuring patients feel listened to and acknowledged [51].

Personalized treatment approaches, tailored to individual patient characteristics, preferences, and values, show substantial promise in pain management. Pharmacogenomics-based interventions for cancer pain [128] and multidisciplinary rehabilitation strategies that combine pharmacological, psychological, and physical therapies have demonstrated improved outcomes for chronic pain patients [129]. Psychological resilience can be enhanced through integrated therapies like cognitive behavioral treatment (CBT), mindfulness, and acceptance approaches [120, 130].

### Barriers to effective pain management

A lack of support and recognition from healthcare staff is a common issue among patients suffering from different types of pain. Insufficient information about proper care and neglect from healthcare lead to feelings of helplessness and anxiety, which worsen pain experiences [75]. Many patients rely on spirituality as a coping mechanism, particularly those with acute and cancer pain [19]. This was more prominent among patients suffering from acute and cancer pain than among those suffering from chronic pain, where other coping strategies were more commonly emphasized, such as finding a balance in their daily life to cope with the pain by for example staying physically active, maintaining hobbies, and/or seeking support from friends and family [70].

Support from friends and family plays a key role in pain management [19, 43]. Some patients, however, fear being perceived as “too whiny” and limit expressing their pain [79]. This delicate balance between seeking support and maintaining independence can hinder effective coping.

Despite various coping mechanisms, no patients described their strategies as ideal. Many had to accept their new lifestyle and develop self-management techniques [59]. Inadequate care forces patients to create their own treatment plans, complicating recovery. The PainSTORY study found that among chronic pain patients dissatisfied with management, 67% experienced no improvement, and 11% reported increased pain over 12 months [10].

### Psychological impact and emotional struggles from pain

The psychological effects of pain are well-documented for chronic, acute, and cancer pain. However, few studies compare their similarities and differences. Psychological stress, anxiety, and depression both result from and exacerbate chronic pain. Pain catastrophizing, where patients anticipate and amplify their pain, intensifies emotional distress and heightens pain perception, creating a vicious cycle [46].

Pain affects concentration, sleep, and memory, leading to a severe psychological burden [46]. Most studies highlight that psychological stress worsens pain perception, reinforcing the bidirectional relationship between pain and mental health.

While fewer studies examine the mental health effects of acute pain, a common theme emerges. Patients describe significant psychological distress, with thoughts of death and death anxiety being prevalent, especially among cancer patients [19]. However, cancer pain, often associated with terminal illness, presents distinct psychological challenges compared to chronic non-malignant pain. Chronic conditions like fibromyalgia or back pain do not carry the same life-threatening implications, influencing patients’ perceptions of their pain. Psychological support must therefore be individualized based on disease type and impact on life.

### Study strengths and limitations

This study followed rigorous methodology, including PROSPERO registration, the PECO framework, and PRISMA guidelines [24, 25]. Expert-assisted searches across multiple databases ensured reliability, with selected articles rated as high to moderate confidence.

A key limitation was the scarcity of data on acute pain experiences. Only five studies met inclusion criteria for acute pain, compared to 44 on chronic pain and 13 on cancer pain. This discrepancy may limit conclusions regarding acute pain and its subjective experience. The limited number of studies available on acute pain restricts comprehensive comparisons and might introduce bias in interpreting differences between acute, chronic, and cancer pain categories. Future research should aim to address this gap. Furthermore, because acute pain is often studied in highly context-specific situations, such as postoperative or intensive care environments, the available evidence may not adequately represent the full spectrum of acute pain experiences. This contextual narrowness could bias interpretations and restrict generalizability, underlining the need for more diverse qualitative studies in acute pain [3, 4].

Moreover, future systematic reviews should consider incorporating studies from additional languages to minimize linguistic exclusion bias, particularly from regions such as Asia, Africa, and Central Europe, where qualitative research is often published in non-included languages. Restricting inclusion to English, Spanish, Portuguese, Greek, and Scandinavian languages may therefore have limited the comprehensiveness of the synthesis. Important qualitative perspectives on pain experiences may be underrepresented, particularly from regions where research is frequently published in other languages such as Mandarin, German, French, or Arabic. This language restriction could thus reduce the representativeness of findings and introduce cultural bias in the synthesized evidence [24, 131].

Even though all studies meeting the inclusion criteria were included, to provide a comprehensive overview of the existing literature, another limitation concerns the broad time span of included studies, dating back to 1999. Pain management practices, healthcare accessibility, and societal attitudes toward pain have changed considerably over the past 25 years. Notably, the IASP revised its definition of chronic pain in 2020 to better capture its multidimensional nature [1]. As a result, older studies may reflect historical contexts and definitions that differ from contemporary standards, which could influence the subjective experiences reported. This temporal heterogeneity should be acknowledged when interpreting findings, as it may affect the synthesis between older and more recent research.

### Clinical implications and generalizability

The clinical implications of this study are of great value, as its findings have the potential to change the current outlook on pain management within healthcare systems. The results from this study suggest that placing more focus on patients’ pain experiences and individualized management strategies results in better pain management. While previous research primarily focuses on each pain type individually, this study compares the different experiences and highlights the importance of individualized assessment for successful pain management. Placing more focus on patients’ pain experiences requires healthcare providers to be more attentive to their patients’ concerns. This means listening to patients’ complaints and experiences with pain, rather than just focusing on symptoms. The findings of this study could be generalized, especially for chronic pain, since sufficient information was collected, and all relevant aspects were taken into account.

To translate these findings into practice, concrete measures should be considered. For healthcare education, this could involve integrating patient narratives and qualitative evidence into medical and nursing curricula, as well as simulation-based training modules in empathic listening and shared decision-making. Policy initiatives might include mandating the systematic use of validated communication tools such as the NRS, with adaptations to reflect patient-centered contexts, and promoting interprofessional collaboration in pain management. Establishing multidisciplinary pain clinics that combine medical, psychological, and social support could also facilitate individualized, holistic care [127–129].

### Future applications

This systematic review can serve as a foundational reference for future studies exploring the subjective experience of pain. The findings emphasize the need for further research to enhance our understanding of the complex nature of pain, its consequences, and patients’ reports about their experience with pain. By expanding the knowledge base in this area, a more comprehensive view of the impact of pain can be developed, which in turn would be crucial for refining pain management strategies. Recommendations for healthcare professional education and policy include enhancing awareness of subjective pain experiences, incorporating patient-centered communication training, and promoting multidisciplinary collaboration to improve pain management outcomes. It is also important to study the long-term effects of pain on social relationships and work life, as well as how well current pain management strategies are working. This would then result in improved pain care and consequently in better quality of life.

## Conclusion

Chronic, acute, and cancer-related pain significantly impact patients’ social, work, and personal lives. Many face inadequate recognition and support from healthcare providers, leading to reliance on self-management strategies and barriers to effective pain relief. Pain further contributes to mental health struggles, including catastrophizing, reduced self-esteem, and identity loss. Further research is necessary to deepen understanding of how different pain types affect patients’ lives and mental health, ultimately improving pain management strategies.

## Supplementary Information

Additional file 1. PRISMA 2020 checklist.

Additional file 2. Search strategy.

Additional file 3. Methodological assessment.

## Data Availability

Data is provided within the manuscript or supplementary information files.

## Abbreviations

CASP: Critical appraisal skills program
GRADE-CERQual: Confidence in the Evidence from Reviews of Qualitative Research
IASP: International Association for the Study of Pain
NRS: Numeric Rating Scale
PECO: Patients, Exposure, Comparison, and Outcome
PRISMA: Preferred Reporting Items for Systematic Reviews and Meta-Analyses
PROSPERO: International Prospective Register of Systematic Reviews
SoQF: Summary of Qualitative Findings
