# Supplementary material for: Living with pain—a systematic review on patients’ subjective experiences
Source: Syst Rev. 2025 Oct 6;14:188. doi: 10.1186/s13643-025-02953-6 (PMC12502253; doi:10.1186/s13643-025-02953-6)
Supplement: Supplementary file 2 — Additional file 2. Search strategy. [file 13643_2025_2953_MOESM2_ESM.docx]

**Supplemental Digital Content 2:** Documentation of search strategies

University Library search consultation group

Date: April 2024

Topic/research question: In adult individuals (P) subjective experience (O) of living with chronic pain (E) or cancer pain (E) in comparison with acute pain (C)

Name of researcher(s): Nikolaos Christidis, Department of Dental Medicine

Librarian(s): Sabina Gillsund

Databases:

1. Medline (Ovid)
2. Embase (embase.com)
3. Cochrane (Wiley)
4. Web of Science (Clarivate Analytics)
5. CINAHL (EBSCO)

Total number of hits:

- Before deduplication: 20,237
- After deduplication: 8,443

1. Medline

| Interface: Ovid MEDLINE(R) ALL  Date of Search: 19 April 2024  Number of hits: 5300  Comment: In Ovid, two or more words are automatically searched as phrases; i.e. no quotation marks are needed | Field labels   - exp/ = exploded MeSH term - / = non exploded MeSH term - .ti,ab,kf. = title, abstract and author keywords - adjx = within x words, regardless of order - * = truncation of word for alternate endings |
| --- | --- |
| Database(s): **Ovid MEDLINE(R) ALL**1946 to April 18, 2024 Search Strategy:   \| **#** \| **Searches** \| **Results** \| \| --- \| --- \| --- \| \| 1 \| Pain/px or Cancer Pain/px or Acute Pain/px or Chronic Pain/px or exp Craniomandibular Disorders/px or Myofascial Pain Syndromes/px or Pain Perception/ \| 23754 \| \| 2 \| pain*.ti,kf. \| 300040 \| \| 3 \| ((acute or cancer or chronic* or long term or longterm or myofascial or orofacial or persist*) adj3 pain*).ab. \| 126911 \| \| 4 \| (craniomandibular adj3 (arthropath* or disease* or disorder* or dysfuncti* or pain* or syndrom*)).ti,ab,kf. \| 705 \| \| 5 \| ((jaw or temporomandibular or tmj) adj3 (arthropath* or disease* or dislocation* or disorder* or dysfuncti* or luxation* or pain* or subluxation* or syndrom*)).ti,ab,kf. \| 15154 \| \| 6 \| or/1-5 \| 369001 \| \| 7 \| "Attitude to health"/ \| 85467 \| \| 8 \| ((experience* or perspective* or perception* or living) adj5 pain*).ti,ab,kf. \| 47811 \| \| 9 \| ((life or lived or men* or patient* or participant* or personal or subjective or wom?n*) adj2 (attitude* or experience* or perception* or perspective* or view*)).ti,ab,kf. \| 319715 \| \| 10 \| ((men* or patient* or participant* or personal or subjective or wom?n*) adj3 (emotion* or feeling*)).ti,ab,kf. \| 33173 \| \| 11 \| or/7-10 \| 455720 \| \| 12 \| exp Qualitative research/ \| 87054 \| \| 13 \| Interviews as topic/ \| 66853 \| \| 14 \| Focus groups/ \| 36950 \| \| 15 \| Narration/ \| 10489 \| \| 16 \| (content analys* or ethnograph* or fieldwork or field work or focus group* or grounded theory or hermeneutic* or informant* or interview* or mixed method* or narration* or narrative* or open question* or participat* observation* or phenomenograph* or phenomenolog* or qualitative* or semi-structured or semistructured or thematic analys*).ti,ab,kf. \| 924971 \| \| 17 \| ((in-depth or indepth or structured or guide or unstructured) adj3 (discussion* or questionnaire*)).ti,ab,kf. \| 36883 \| \| 18 \| or/12-17 \| 967729 \| \| 19 \| 6 and 11 and 18 \| 5839 \| \| 20 \| (exp Child/ or exp Infant/ or exp Adolescent/ or (child? or children* or childhood or adolescen* or infant* or neonat* or juvenile* or p?ediatric*).ti.) not (exp Adult/ or (adult* or female* or male* or middle age* or older* or elder* or old people* or old person* or men* or man or wom?n*).ti.) \| 2382306 \| \| 21 \| 19 not 20 \| 5310 \| \| 22 \| exp animals/ not humans.sh. \| 5213003 \| \| 23 \| 21 not 22 \| 5300 \| | |

2. Embase

| Interface: embase.com  Date of Search: 19 April 2024  Number of hits: 5592  Comment: Emtree is the controlled vocabulary in Embase | Field labels   - /exp = exploded Emtree term - /de = non exploded Emtree term - ti,ab,kw = title, abstract and author keywords - NEAR/x = within x words, regardless of order - * = truncation of word for alternate endings |
| --- | --- |
| \| **No.** \| **Query** \| **Results** \| \| --- \| --- \| --- \| \| #26 \| #24 NOT #25 \| 5592 \| \| #25 \| #24 AND ('Conference Abstract'/it OR 'Conference Review'/it) \| 1598 \| \| #24 \| #23 NOT ([animals]/lim NOT [humans]/lim) \| 7190 \| \| #23 \| #21 NOT #22 \| 7211 \| \| #22 \| ('juvenile'/exp OR child$:ti OR children*:ti OR childhood:ti OR adolescen*:ti OR infant*:ti OR neonat*:ti OR juvenile*:ti OR p$ediatric*:ti) NOT ('adult'/exp OR adult*:ti OR female*:ti OR male*:ti OR 'middle age*':ti OR older*:ti OR elder*:ti OR 'old people*':ti OR 'old person*':ti OR men*:ti OR man:ti OR wom$n*:ti) \| 3069890 \| \| #21 \| #10 AND #15 AND #20 \| 7830 \| \| #20 \| #16 OR #17 OR #18 OR #19 \| 1244281 \| \| #19 \| (('in depth' OR indepth OR structured OR guide OR unstructured) NEAR/3 (discussion* OR questionnaire*)):ti,ab,kw \| 48664 \| \| #18 \| 'content analys*':ti,ab,kw OR ethnograph*:ti,ab,kw OR fieldwork:ti,ab,kw OR 'field work':ti,ab,kw OR 'focus group*':ti,ab,kw OR 'grounded theory':ti,ab,kw OR hermeneutic*:ti,ab,kw OR informant*:ti,ab,kw OR interview*:ti,ab,kw OR 'mixed method*':ti,ab,kw OR narration*:ti,ab,kw OR narrative*:ti,ab,kw OR 'open question*':ti,ab,kw OR 'participat* observation*':ti,ab,kw OR phenomenograph*:ti,ab,kw OR phenomenolog*:ti,ab,kw OR qualitative*:ti,ab,kw OR 'semi structured':ti,ab,kw OR semistructured:ti,ab,kw OR 'thematic analys*':ti,ab,kw \| 1135841 \| \| #17 \| 'interview'/exp \| 388140 \| \| #16 \| 'qualitative research'/exp \| 126689 \| \| #15 \| #11 OR #12 OR #13 OR #14 \| 666457 \| \| #14 \| ((men* OR patient* OR participant* OR personal OR subjective OR wom$n*) NEAR/3 (emotion* OR feeling*)):ti,ab,kw \| 46874 \| \| #13 \| ((life OR lived OR men* OR patient* OR participant* OR personal OR subjective OR wom$n*) NEAR/2 (attitude* OR experience* OR perception* OR perspective* OR view*)):ti,ab,kw \| 456297 \| \| #12 \| ((experience* OR perspective* OR perception* OR living) NEAR/5 pain*):ti,ab,kw \| 67725 \| \| #11 \| 'attitude to health'/de \| 135858 \| \| #10 \| #1 OR #2 OR #3 OR #4 OR #5 OR #6 OR #7 OR #8 OR #9 \| 547717 \| \| #9 \| ((jaw OR temporomandibular OR tmj) NEAR/3 (arthropath* OR disease* OR dislocation* OR disorder* OR dysfuncti* OR luxation* OR pain* OR subluxation* OR syndrom*)):ti,ab,kw \| 17348 \| \| #8 \| (craniomandibular NEAR/3 (arthropath* OR disease* OR disorder* OR dysfuncti* OR pain* OR syndrom*)):ti,ab,kw \| 808 \| \| #7 \| ((acute OR cancer OR chronic* OR 'long term' OR longterm OR myofascial OR orofacial OR persist*) NEAR/3 pain*):ab \| 188158 \| \| #6 \| pain*:ti,kw \| 415827 \| \| #5 \| 'nociception'/mj \| 15363 \| \| #4 \| 'temporomandibular joint disorder'/mj OR 'myofascial pain'/mj \| 17139 \| \| #3 \| 'chronic pain'/exp/mj \| 36159 \| \| #2 \| 'cancer pain'/mj \| 11606 \| \| #1 \| 'pain'/mj \| 106687 \| | |

3. Cochrane Library

| Interface: Wiley  Date of Search: 19 April 2024  Number of hits: 915 | Field labels   - ti,ab,kw = title, abstract and author keywords - NEAR/x = within x words, regardless of order - * = truncation of word for alternate endings |
| --- | --- |
| \| **ID** \| **Search** \| **Hits** \| \| --- \| --- \| --- \| \| #1 \| [mh ^Pain] OR [mh ^"Cancer Pain"] OR [mh ^"Acute Pain"] OR [mh ^"Chronic Pain"] OR [mh "Craniomandibular Disorders"] OR [mh ^"Myofascial Pain Syndromes"] OR [mh ^"Pain Perception"] \| 25972 \| \| #2 \| pain*:ti,kw \| 165695 \| \| #3 \| ((acute:ab OR cancer:ab OR chronic*:ab OR "long term":ab OR longterm:ab OR myofascial:ab OR orofacial:ab OR persist*:ab) NEAR/3 pain*:ab) \| 30232 \| \| #4 \| (craniomandibular:ti,ab,kw NEAR/3 (arthropath*:ti,ab,kw OR disease*:ti,ab,kw OR disorder*:ti,ab,kw OR dysfuncti*:ti,ab,kw OR pain*:ti,ab,kw OR syndrom*:ti,ab,kw)) \| 74 \| \| #5 \| ((jaw:ti,ab,kw OR temporomandibular:ti,ab,kw OR tmj:ti,ab,kw) NEAR/3 (arthropath*:ti,ab,kw OR disease*:ti,ab,kw OR dislocation*:ti,ab,kw OR disorder*:ti,ab,kw OR dysfuncti*:ti,ab,kw OR luxation*:ti,ab,kw OR pain*:ti,ab,kw OR subluxation*:ti,ab,kw OR syndrom*:ti,ab,kw)) \| 2652 \| \| #6 \| #1 OR #2 OR #3 OR #4 OR #5 \| 172142 \| \| #7 \| [mh ^"Attitude to health"] \| 3462 \| \| #8 \| ((experience*:ti,ab,kw OR perspective*:ti,ab,kw OR perception*:ti,ab,kw OR living:ti,ab,kw) NEAR/5 pain*:ti,ab,kw) \| 15289 \| \| #9 \| ((life:ti,ab,kw OR lived:ti,ab,kw OR men*:ti,ab,kw OR patient*:ti,ab,kw OR participant*:ti,ab,kw OR personal:ti,ab,kw OR subjective:ti,ab,kw OR wom?n*:ti,ab,kw) NEAR/2 (attitude*:ti,ab,kw OR experience*:ti,ab,kw OR perception*:ti,ab,kw OR perspective*:ti,ab,kw OR view*:ti,ab,kw)) \| 45508 \| \| #10 \| ((men*:ti,ab,kw OR patient*:ti,ab,kw OR participant*:ti,ab,kw OR personal:ti,ab,kw OR subjective:ti,ab,kw OR wom?n*:ti,ab,kw) NEAR/3 (emotion*:ti,ab,kw OR feeling*:ti,ab,kw)) \| 6126 \| \| #11 \| #7 OR #8 OR #9 OR #10 \| 64871 \| \| #12 \| [mh "Qualitative research"] \| 2287 \| \| #13 \| [mh ^"Interviews as topic"] \| 2511 \| \| #14 \| [mh ^"Focus groups"] \| 1050 \| \| #15 \| [mh ^Narration] \| 298 \| \| #16 \| (("content" NEXT analys*):ti,ab,kw OR ethnograph*:ti,ab,kw OR fieldwork:ti,ab,kw OR "field work":ti,ab,kw OR ("focus" NEXT group*):ti,ab,kw OR "grounded theory":ti,ab,kw OR hermeneutic*:ti,ab,kw OR informant*:ti,ab,kw OR interview*:ti,ab,kw OR ("mixed" NEXT method*):ti,ab,kw OR narration*:ti,ab,kw OR narrative*:ti,ab,kw OR ("open" NEXT question*):ti,ab,kw OR (participat* NEXT observation*):ti,ab,kw OR phenomenograph*:ti,ab,kw OR phenomenolog*:ti,ab,kw OR qualitative*:ti,ab,kw OR semi-structured:ti,ab,kw OR semistructured:ti,ab,kw OR ("thematic" NEXT analys*):ti,ab,kw) \| 73739 \| \| #17 \| ((in-depth:ti,ab,kw OR indepth:ti,ab,kw OR structured:ti,ab,kw OR guide:ti,ab,kw OR unstructured:ti,ab,kw) NEAR/3 (discussion*:ti,ab,kw OR questionnaire*:ti,ab,kw)) \| 2708 \| \| #18 \| #12 OR #13 OR #14 OR #15 OR #16 OR #17 \| 75110 \| \| #19 \| #6 AND #11 AND #18 \| 1288 \| \| #20 \| ([mh Child] OR [mh Infant] OR [mh Adolescent] OR (child?:ti OR children*:ti OR childhood:ti OR adolescen*:ti OR infant*:ti OR neonat*:ti OR juvenile*:ti OR p?ediatric*:ti)) NOT ([mh Adult] OR (adult*:ti OR female*:ti OR male*:ti OR ("middle" NEXT age*):ti OR older*:ti OR elder*:ti OR ("old" NEXT people*):ti OR ("old" NEXT person*):ti OR men*:ti OR man:ti OR wom?n*:ti)) \| 165767 \| \| #21 \| #19 NOT #20 \| 1199 \| \| #22 \| #21 NOT (clinicaltrials or trialsearch):so \| 915 \| | |

4. Web of Science Core Collection

| Interface: Clarivate Analytics  Editions = A&HCI , ESCI , SCI-EXPANDED , SSCI  Date of Search: 19 April 2024  Number of hits: 4840 | Field labels   - TS/Topic = title, abstract, author keywords and Keywords Plus - NEAR/x = within x words, regardless of order - * = truncation of word for alternate endings   Note: the *Exact search*-function was used for all the searches |
| --- | --- |
| \| **#** \| **Search Query** \| **Results** \| \| --- \| --- \| --- \| \| 1 \| TI=pain* OR AK=pain* \| 398550 \| \| 2 \| AB=((acute OR cancer OR chronic* OR "long term" OR longterm OR myofascial OR orofacial OR persist*) NEAR/2 pain*) \| 106394 \| \| 3 \| TS=(craniomandibular NEAR/2 (arthropath* OR disease* OR disorder* OR dysfuncti* OR pain* OR syndrom*)) \| 823 \| \| 4 \| TS=((jaw OR temporomandibular OR tmj) NEAR/3 (arthropath* OR disease* OR dislocation* OR disorder* OR dysfuncti* OR luxation* OR pain* OR subluxation* OR syndrom*)) \| 13822 \| \| 5 \| #1 OR #2 OR #3 OR #4 \| 446167 \| \| 6 \| TS=((experience* OR perspective* OR perception* OR living) NEAR/4 pain*) \| 44484 \| \| 7 \| TS=((life OR lived OR men* OR patient* OR participant* OR personal OR subjective OR wom$n*) NEAR/1 (attitude* OR experience* OR perception* OR perspective* OR view*)) \| 362506 \| \| 8 \| TS=((men* OR patient* OR participant* OR personal OR subjective OR wom$n*) NEAR/2 (emotion* OR feeling*)) \| 38452 \| \| 9 \| #6 OR #7 OR #8 \| 427682 \| \| 10 \| TS=("content analys*" OR ethnograph* OR fieldwork OR "field work" OR "focus group*" OR "grounded theory" OR hermeneutic* OR informant* OR interview* OR "mixed method*" OR narration* OR narrative* OR "open question*" OR "participat* observation*" OR phenomenograph* OR phenomenolog* OR qualitative* OR semi-structured OR semistructured OR "thematic analys*") \| 1810575 \| \| 11 \| TS=((in-depth OR indepth OR structured OR guide OR unstructured) NEAR/2 (discussion* OR questionnaire*)) \| 45032 \| \| 12 \| #10 OR #11 \| 1834119 \| \| 13 \| #5 AND #9 AND #12 \| 5351 \| \| 14 \| TI=(child$ OR children* OR childhood OR adolescen* OR infant* OR neonat* OR juvenile* OR p$ediatric*) NOT TI=(adult* OR female* OR male* OR "middle age*" OR older* OR elder* OR "old people*" OR "old person*" OR men* OR man OR wom$n*) \| 1814437 \| \| 15 \| #13 NOT #14 and Meeting Abstract (Exclude – Document Types) \| 4840 \| | |

5. Cinahl

| Interface: Ebsco  Date of Search: 19 April 2024  Number of hits: 3590 | Field labels   - MH+ = exploded Cinahl Heading - MH = non exploded Cinahl Heading - TI = title - AB = abstract - Nx = within x words, regardless of order - * = truncation of word for alternate endings   Note: sometimes “quotation marks” are needed for single search terms to avoid automatic term mapping (lemmatization)1 |
| --- | --- |
| \| **#** \| **Query** \| **Results** \| \| --- \| --- \| --- \| \| S25 \| S23 NOT S24 \| 3,590 \| \| S24 \| ((MH "Child+") OR (MH "Adolescence+") OR TI ((child# OR children* OR childhood OR adolescen* OR infant* OR neonat* OR juvenile* OR p#ediatric*))) NOT ((MH "Adult+") OR TI ((adult* OR female* OR male* OR "middle age*" OR older* OR elder* OR "old people*" OR "old person*" OR men* OR man OR wom#n*))) \| 793,931 \| \| S23 \| S10 AND S15 AND S22 \| 3,997 \| \| S22 \| S16 OR S17 OR S18 OR S19 OR S20 OR S21 \| 575,321 \| \| S21 \| TI ( ((in-depth OR indepth OR structured OR guide OR unstructured) N2 (discussion* OR questionnaire*)) ) OR AB ( ((in-depth OR indepth OR structured OR guide OR unstructured) N2 (discussion* OR questionnaire*)) ) \| 15,234 \| \| S20 \| TI ( ("content analys*" OR ethnograph* OR fieldwork OR "field work" OR "focus group*" OR "grounded theory" OR hermeneutic* OR informant* OR interview* OR "mixed method*" OR narration* OR narrative* OR "open question*" OR "participat* observation*" OR phenomenograph* OR phenomenolog* OR qualitative* OR semi-structured OR semistructured OR "thematic analys*") ) OR AB ( ("content analys*" OR ethnograph* OR fieldwork OR "field work" OR "focus group*" OR "grounded theory" OR hermeneutic* OR informant* OR interview* OR "mixed method*" OR narration* OR narrative* OR "open question*" OR "participat* observation*" OR phenomenograph* OR phenomenolog* OR qualitative* OR semi-structured OR semistructured OR "thematic analys*") ) \| 445,953 \| \| S19 \| (MH "Narratives+") \| 21,421 \| \| S18 \| (MH "Focus Groups") \| 52,309 \| \| S17 \| (MH "Interviews+") \| 265,014 \| \| S16 \| (MH "Qualitative Studies+") \| 193,071 \| \| S15 \| S11 OR S12 OR S13 OR S14 \| 250,631 \| \| S14 \| TI ( ((men* OR patient* OR participant* OR personal OR subjective OR wom#n*) N3 (emotion* OR feeling*)) ) OR AB ( ((men* OR patient* OR participant* OR personal OR subjective OR wom#n*) N3 (emotion* OR feeling*)) ) \| 21,451 \| \| S13 \| TI ( ((life OR lived OR men* OR patient* OR participant* OR personal OR subjective OR wom#n*) N1 (attitude* OR experience* OR perception* OR perspective* OR view*)) ) OR AB ( ((life OR lived OR men* OR patient* OR participant* OR personal OR subjective OR wom#n*) N1 (attitude* OR experience* OR perception* OR perspective* OR view*)) ) \| 147,406 \| \| S12 \| TI ( ((experience* OR perspective* OR perception* OR living) N4 pain*) ) OR AB ( ((experience* OR perspective* OR perception* OR living) N4 pain*) ) \| 20,790 \| \| S11 \| (MH "Attitude to Health") OR (MH "Life Experiences") \| 90,301 \| \| S10 \| S1 OR S2 OR S3 OR S4 OR S5 OR S6 OR S7 OR S8 OR S9 \| 165,557 \| \| S9 \| TI ( ((jaw OR temporomandibular OR tmj) N2 (arthropath* OR disease* OR dislocation* OR disorder* OR dysfuncti* OR luxation* OR pain* OR subluxation* OR syndrom*)) ) OR AB ( ((jaw OR temporomandibular OR tmj) N2 (arthropath* OR disease* OR dislocation* OR disorder* OR dysfuncti* OR luxation* OR pain* OR subluxation* OR syndrom*)) ) \| 4,913 \| \| S8 \| TI ( (craniomandibular N2 (arthropath* OR disease* OR disorder* OR dysfuncti* OR pain* OR syndrom*)) ) OR AB ( (craniomandibular N2 (arthropath* OR disease* OR disorder* OR dysfuncti* OR pain* OR syndrom*)) ) \| 78 \| \| S7 \| AB (((acute OR cancer OR chronic* OR "long term" OR longterm OR myofascial OR orofacial OR persist*) N2 pain*)) \| 46,577 \| \| S6 \| TI pain* \| 142,884 \| \| S5 \| (MH "Myofascial Pain Syndromes+/PF") \| 90 \| \| S4 \| (MH "Craniomandibular Disorders+/PF") \| 313 \| \| S3 \| (MH "Cancer Pain/PF") \| 271 \| \| S2 \| (MH "Chronic Pain/PF") \| 3,184 \| \| S1 \| (MH "Pain/PF") \| 6,406 \| | |
