# Supplementary material for: Living with pain—a systematic review on patients’ subjective experiences
Source: Syst Rev. 2025 Oct 6;14:188. doi: 10.1186/s13643-025-02953-6 (PMC12502253; doi:10.1186/s13643-025-02953-6)
Supplement: Supplementary file 3 — Additional file 3. Methodological assessment. [file 13643_2025_2953_MOESM3_ESM.docx]

**Supplemental Digital Content 3:** Methodological assessment of each individual article of the 89 articles included in the systematic review.

| **Author(s), Year** | **Was there a clear statement of the aims of the research?** | **Is a qualitative methodology appropriate?** | **Was the research design appropriate to address the aims**  **of the research?** | **Was the recruitment strategy appropriate to the aims of**  **the research?** | **Was the data collected in a way that addressed the**  **research issue?** | **Has the relationship between researcher and participants been adequately considered?** | **Have ethical issues been taken into consideration?** | **Was the data analysis sufficiently rigorous?** | **Is there a clear statement of findings?** | **How valuable is the research?** |
| --- | --- | --- | --- | --- | --- | --- | --- | --- | --- | --- |
| 1. Aegler & Satink 2009 | Yes | Yes | Yes | Yes | Yes | Yes | Yes | Yes | Yes | Yes |
| 1. Antunovich et al. 2021 | Yes | Yes | Yes | Yes | Yes | Yes | Yes | Yes | Yes | Yes |
| 1. Armoogum et al. 2023 | Yes | Yes | Yes | Yes | Yes | Yes | Yes | Yes | Yes | Yes |
| 1. Blomqvist & Edberg 2002 | Yes | Yes | Yes | Yes | Yes | Yes | Yes | Yes | Yes | Yes |
| 1. Bowman 1991 | No | Yes | Can't tell | Can't tell | Can't tell | No | No | No | Yes | Yes |
| 1. Campbell & Cramb 2008 | Yes | Yes | Yes | Can't tell | Yes | No | Yes | Yes | Yes | Yes |
| 1. Christensen et al. 2023 | Yes | Yes | Yes | Yes | Yes | Yes | Yes | Yes | Yes | Yes |
| 1. Clarke & Iphofen 2007 | Yes | Yes | Yes | Yes | Yes | No | No | Can't tell | Yes | Yes |
| 1. Clarke et al. 2012 | Yes | Yes | Yes | Yes | Yes | Yes | Yes | Yes | Yes | Yes |
| 1. Crowe et al. 2010 | Yes | Yes | Yes | Yes | Yes | Yes | Yes | Yes | Yes | Yes |
| 1. Cummings et al. 2017 | Yes | Yes | Yes | Yes | Yes | No | Yes | Yes | Yes | Yes |
| 1. Cunningham & Jillings 2006 | Yes | Yes | Yes | Can't tell | Yes | Yes | No | Yes | Yes | Yes |
| 1. De Souza & Frank 2007 | Yes | Yes | Yes | Can’t tell | Yes | No | Yes | Yes | Yes | Yes |
| 1. De Souza & Frank 2011 | Yes | Yes | Yes | Can’t tell | Yes | No | Yes | Yes | Yes | Yes |
| 1. Dow et al. 2012 | Yes | Yes | Yes | Yes | Yes | Can't tell | Yes | Yes | Yes | Yes |
| 1. Duggleby 2000 | Yes | Yes | Yes | Yes | Yes | No | Yes | Yes | Yes | Yes |
| 1. Dunham et al. 2017 | Yes | Yes | Yes | No | No | No | Yes | No | Yes | Yes |
| 1. El-Haddad et al. 2018 | Yes | Yes | Yes | Yes | Yes | No | Yes | Yes | Yes | Yes |
| 1. Erol et al. 2018 | Yes | Yes | Yes | No | Yes | No | Yes | Yes | Yes | Yes |
| 1. Esson et al. 2020 | Yes | Yes | Yes | Yes | Yes | Yes | Yes | Yes | Yes | Yes |
| 1. Fisher et al. 2007 | Yes | Yes | Yes | Yes | Yes | Yes | Yes | Yes | Yes | Yes |
| 1. Gillsjö et al. 2021 | Yes | Yes | Yes | No | Yes | Yes | Yes | Yes | Yes | Yes |
| 1. Gulseren & Kelloway 2021 | Yes | Yes | Yes | No | Yes | No | Yes | Yes | Yes | Yes |
| 1. Hallberg & Carlsson 2000 | Yes | Yes | Yes | Yes | Yes | Yes | Yes | Yes | Yes | Yes |
| 1. Hassankhani et al. 2023 | Yes | Yes | Yes | Yes | Yes | Yes | Yes | Yes | Yes | Yes |
| 1. Hazaveh & Hovey 2018 | Yes | Yes | Yes | No | Yes | Yes | Yes | Yes | Yes | Yes |
| 1. Hellerstedt-Börjesson et al. 2016 | Yes | Yes | Yes | Yes | Yes | Yes | Yes | Yes | Yes | Yes |
| 1. Henriksson 1995 | Yes | Yes | Yes | Can't tell | Yes | No | No | Yes | Yes | Yes |
| 1. Hervik et al. 2023 | Yes | Yes | Yes | Yes | Yes | Yes | Yes | Yes | Yes | Yes |
| 1. Horment-Lara et al. 2022 | Yes | Yes | Yes | Yes | Yes | Yes | Yes | Yes | Yes | Yes |
| 1. Hovind et al. 2013 | Yes | Yes | Yes | Can’t tell | Yes | Yes | Yes | Yes | Yes | Yes |
| 1. Hunsche et al. 2023 | Yes | Yes | Yes | Can’t tell | No | Can't tell | Yes | Yes | Yes | Yes |
| 1. Hylén et al. 2020 | Yes | Yes | Yes | Yes | Yes | Yes | Yes | Yes | Yes | Yes |
| 1. Igwesi-Chidobe et al. 2017 | Yes | Yes | Yes | Yes | Yes | Yes | Yes | Yes | Yes | Yes |
| 1. Ilgunas et al. 2023 | Yes | Yes | Yes | Yes | Yes | Yes | Yes | Yes | Yes | Yes |
| 1. Jerlock et al. 2005 | Yes | Yes | Yes | Yes | Yes | No | Yes | Yes | Yes | Yes |
| 1. Jones et al. 2023 | Yes | Yes | Yes | Yes | Yes | Yes | Yes | Yes | Yes | Yes |
| 1. Juuso et al. 2011 | Yes | Yes | Yes | Yes | Yes | No | Yes | Yes | Yes | Yes |
| 1. Juuso et al. 2016 | Yes | Yes | Yes | Yes | Yes | Yes | Yes | Yes | Yes | Yes |
| 1. Kurz & Hebron 2024 | Yes | Yes | Yes | Yes | Yes | Yes | Yes | Yes | Yes | Yes |
| 1. Kuuppelomäki & Lauri 1998 | Yes | Yes | Yes | Yes | No | No | No | Yes | Yes | Yes |
| 1. Kwon & Kim 2021 | Yes | Yes | Yes | Yes | Yes | Yes | Yes | Yes | Yes | Yes |
| 1. Larsson & Wijk 2007 | Yes | Yes | Yes | Yes | Yes | No | Yes | Yes | Yes | Yes |
| 1. Lavie-Ajayi et al. 2012 | Yes | Yes | Yes | Yes | Yes | No | Yes | Yes | Yes | Yes |
| 1. Law et al. 2019 | Yes | Yes | Yes | Yes | Yes | Yes | Yes | Yes | Yes | Yes |
| 1. Lundberg et al. 2007 | Yes | Yes | Yes | Yes | Yes | Yes | Yes | Yes | Yes | Yes |
| 1. Mackichan et al. 2013 | Yes | Yes | Yes | Yes | Yes | No | Yes | Yes | Yes | Yes |
| 1. Makris et al. 2017 | Yes | Yes | Yes | Yes | Yes | No | Yes | Yes | Yes | Yes |
| 1. Mannerkorpi et al. 1999 | Yes | Yes | Yes | Yes | Yes | Yes | Yes | Yes | Yes | Yes |
| 1. Martin 1989 | Yes | Yes | Yes | Yes | Yes | No | Yes | Yes | Yes | Yes |
| 1. Martin et al. 2015 | Yes | Yes | Yes | Yes | Yes | No | Yes | Yes | Yes | Yes |
| 1. McCarthy et al. 2021 | Yes | Yes | Yes | Yes | Yes | Yes | No | Yes | Yes | Yes |
| 1. McHugh & Thoms 2001 | Yes | Yes | Yes | Yes | Yes | Yes | Yes | Yes | Yes | Yes |
| 1. Michaëlis et al. 2015 | Yes | Yes | Yes | Yes | Yes | No | Yes | Yes | Yes | Yes |
| 1. Miles et al. 2005 | Yes | Yes | Yes | Yes | Yes | No | Yes | Yes | Yes | Yes |
| 1. Mok & Lee 2008 | Yes | Yes | Yes | Yes | Yes | No | Yes | Yes | Yes | Yes |
| 1. Nielsen et al. 2013 | Yes | Yes | Yes | Yes | Yes | Yes | Yes | Yes | Yes | Yes |
| 1. Nilmanat et al. 2010 | Yes | Yes | Yes | Yes | Yes | No | Yes | Yes | Yes | No |
| 1. Nilsen & Anderssen 2014 | Yes | Yes | Yes | Yes | Yes | No | Yes | Yes | Yes | Yes |
| 1. Ojala et al. 2015 | Yes | Yes | Yes | Yes | Yes | No | Yes | Yes | Yes | Yes |
| 1. Orujlu et al. 2022 | Yes | Yes | Yes | Yes | Yes | Yes | Yes | Yes | Yes | Yes |
| 1. Paulson et al. 2002 | Yes | Yes | Yes | Yes | Yes | Yes | Yes | Yes | Yes | No |
| 1. Paulson et al. 2002 | Yes | Yes | Yes | Yes | Yes | No | Yes | Yes | Yes | No |
| 1. Penney et al. 2017 | Yes | Yes | Yes | Yes | Yes | Yes | Yes | Yes | Yes | Yes |
| 1. Roberto & Reynolds 2002 | Yes | Yes | Yes | Can't tell | Yes | No | No | Yes | Yes | Yes |
| 1. Robinson & Maree 2024 | Yes | Yes | Yes | Yes | Yes | Can't tell | Yes | Yes | Yes | Yes |
| 1. Sakyi et al. 2024 | Yes | Yes | Yes | Yes | Yes | Yes | Yes | Yes | Yes | Yes |
| 1. Sallinen & Mengshoel 2019 | Yes | Yes | Yes | Yes | Yes | Yes | Yes | Yes | Yes | Yes |
| 1. Seers 1996 | Yes | Yes | Yes | Yes | Yes | No | No | Yes | Yes | Yes |
| 1. Simonsen-Rehn et al. 2000 | Yes | Yes | Yes | Yes | Yes | No | Yes | Yes | Yes | Yes |
| 1. Sofaer et al. 2005 | Yes | Yes | Yes | Yes | Yes | No | Yes | Yes | Yes | Yes |
| 1. Stensland & Sanders 2018 | Yes | Yes | Yes | Yes | Yes | No | No | Yes | Yes | Yes |
| 1. Stensland & Sanders 2018 | Yes | Yes | Yes | Yes | Yes | No | No | Yes | Yes | Yes |
| 1. Strang 1992 | Yes | Yes | Yes | Can't tell | Yes | No | No | Yes | Yes | Yes |
| 1. Sturge-Jacobs 2002 | Yes | Yes | Yes | Yes | Yes | No | Yes | Yes | Yes | Yes |
| 1. Söderberg et al. 1999 | Yes | Yes | Yes | Yes | Yes | Can't tell | Yes | Yes | Yes | Yes |
| 1. Voorhees 2023 | Yes | No | Yes | No | Yes | No | No | Yes | Yes | Can't tell |
| 1. Wade 2003 | Yes | Yes | Yes | Yes | Yes | Yes | No | Yes | Yes | Yes |
| 1. Walker et al. 1999 | Yes | Yes | Yes | Yes | Yes | Yes | Yes | Yes | Yes | Yes |
| 1. Webber et al. 2011 | Yes | Yes | Yes | Yes | Yes | Can't tell | Yes | Yes | Yes | Yes |
| 1. Westergården et al. 2021 | Yes | Yes | Yes | Yes | Yes | No | Yes | Yes | Yes | Yes |
| 1. Wolf 2006 | Yes | Yes | Yes | Yes | Yes | Can't tell | Yes | Yes | Yes | Yes |
| 1. Wolf et al. 2008 | Yes | Yes | Yes | Yes | Yes | Can't tell | Yes | Yes | Yes | Yes |
| 1. Wolf et al. 2016 | Yes | Yes | Yes | Yes | Yes | Can't tell | Yes | Yes | Yes | Yes |
| 1. Wong et al. 2008 | Yes | Cant tell | Yes | No | Yes | No | Yes | Yes | Yes | Yes |
| 1. Xia et al. 2024 | Yes | Yes | Yes | Yes | Yes | Yes | Yes | Yes | Yes | Yes |
| 1. Xu et al. 2019 | Yes | Yes | Yes | Yes | Yes | Yes | Yes | Yes | Yes | Yes |
| 1. Yeowell et al. 2021 | Yes | Yes | Yes | Yes | Yes | Yes | Yes | Yes | Yes | Yes |
| 1. Yildizeli Topcu 2018 | Yes | Yes | Yes | Yes | Yes | Yes | No | Yes | Yes | Yes |
